# Comparison and Investigation of Exosomes from Human Amniotic Fluid Stem Cells and Human Breast Milk in Alleviating Neonatal Necrotizing Enterocolitis

**DOI:** 10.1007/s12015-022-10470-5

**Published:** 2022-11-16

**Authors:** Xiaohan Hu, Rui Zhang, Hansi Liang, Jingnan An, Yuan Yang, Jie Huo, Zhenjiang Chen, Wei Quan, Lu Jiang, Cancan Li, Jian Li, Fang Li, Yunyun Xu, Xueping Zhu

**Affiliations:** 1 Institute of Pediatric, Children’s Hospital of Soochow University, Suzhou City, Jiangsu Province China; 2 Department of Neonatology, Children’s Hospital of Soochow University, Suzhou City, Jiangsu Province China; 3 Jiangsu Key Laboratory of Gastrointestinal Tumor Immunology, The First Affiliated Hospital of Soochow University, Suzhou, Jiangsu Province China; 4 Institute of Blood and Marrow Transplantation, The First Affiliated Hospital of Soochow University, Suzhou, Jiangsu Province China; 5 Department of Osteology, Honghui Hospital, Xi’an Jiaotong University, Xi’an City, Shaanxi Province China; 6 Department of Osteology, The Second Affiliated Hospital of Soochow University, Suzhou, Jiangsu Province China; 7 Department of Neonatology, Yangzhou Maternity and Child Health Care Hospital, Yangzhou, Jiangsu Province China; 8 Department of Nephrology and Immunology, Children’s Hospital of Soochow University, Suzhou, Jiangsu Province, China; 9 Department of Human Anatomy and Histology & Embryology, Soochow University, Suzhou, Jiangsu Province, China

**Keywords:** Exosomes, Human amniotic fluid stem cells, Human breast milk, Neonatal necrotizing enterocolitis, RNA-Seq

## Abstract

In view of the devastating impact of neonatal necrotizing enterocolitis (NEC) on newborns, the research on its intervention is particularly important. Although exosomes from human amniotic fluid stem cells (AFSC) and human breast milk (HBM) can protect against NEC, their mechanisms remain unclear. Here, we intend to compare the intervention effects of two types of exosomes on NEC mouse model and reveal their respective regulatory mechanisms. In general, both AFSC-derived exosomes (AFSC-exos) and HBM-derived exosomes (HBM- exos) can alleviate NEC- associated intestinal injury, significantly reduce NEC score, and reduce systemic and ileal inflammation and NEC related brain injury during experimental NEC. However, the mode and mechanism of action of the two sources of exosomes were not identical. In vivo, the number of ileal crypts was more significantly restored after HBM-exos intervention than AFSC-exos, and in vitro, HBM-exos preferentially inhibited the inflammatory response of intestinal epithelial cells (IECs), whereas AFSC-exos preferentially regulated the migration of IECs. Mechanistically, GO and KEGG analyses revealed the different therapeutic mechanisms of AFSC-exos and HBM-exos in NEC. Taken together, our results illustrate that AFSC-exos and HBM-exos reduce the severity of experimental NEC and intestinal damage through different mechanisms, supporting the potential of cell-free or breast milk free exosome therapy for NEC.

**Graphical Abstract:**

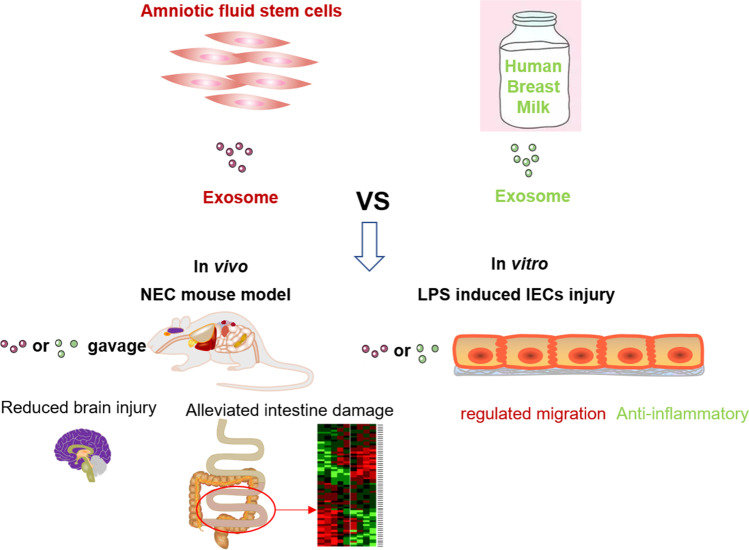

**Supplementary Information:**

The online version contains supplementary material available at 10.1007/s12015-022-10470-5.

## Introduction

Neonatal necrotizing enterocolitis (NEC) can have devastating effects on newborns, especially premature infants. Currently, treatment is limited to non-specific nutritional support and surgical treatment [1]. In traditional treatment, when nutritional support cannot control the illness, NEC will cause secondary deterioration. The occurrence of acute lethal clinical manifestations, such as peritonitis and abdominal gas, requires emergency surgical resection of the long intestine, which increases the total mortality rate to 30% [2]. Some of the children who are treated also develop a range of complications, such as short bowel syndrome [2], which further endangers the long-term survival of the children. Therefore, there is an urgent need to explore new therapeutic methods to effectively control the occurrence and development of NEC and further explore its protective mechanism, with the aim of improving the prognosis of children with NEC.

Human amniotic fluid stem cells (AFSCs) and breast milk (HBM) are hot topics in NEC treatment, and their effects on NEC have been confirmed [3, 4]. Studies have shown that amniotic fluid-derived mesenchymal stem cells and neural stem cells can protect NEC intestines from injury [5]. Randomized trials demonstrated that breast-feeding reduced the incidence of NEC in preterm infants compared with formula feeding [6, 7].

Human amniotic fluid stem cells (AFSCs) and breast milk (HBM) have therapeutic effects on NEC model. With the deepening of research, it was found that their therapy mainly works through paracrine manner. Therefore, we and other scholars consider using exosomes secreted by stem cells as alternative therapy. Exosomes are nanoscale vesicles containing biologically active molecules, such as nucleic acids, proteins, and lipids, and comprise unique lipid bilayers that protect them from RNA enzymes and proteases, meaning they are ideal for treating disease. Exosomes have been proven to have biological functions in inflammation, tumors, and autoimmune diseases [8–10]. Our previous research showed AFSCs can create a moderate inflammation microenvironment to promote wound healing [11]; however, the role of exosomes secreted by AFSCs (AFSC-exos) in intestinal inflammation has not been explored. We have also demonstrated that HBM-derived exosomes (HBM-exos) might help maintain the intestinal epithelial barrier integrity during NEC [12]; however, the specific mechanism requires further exploration.

In this study, we compared the ability of the two types of exosomes to improve experimentally NEC- induced intestinal injury, restore intestinal regeneration, inhibit inflammation, and improve NEC-related complications, thus treating NEC. Moreover, RNA sequencing was used to gain more insight into the potential treatment mechanism of AFSC-exos and HBM-exos in NEC- associated intestinal injury in mice. Thus, our work reveals the differential function and mechanism of AFSC-exos and HBM-exos in reducing the severity of experimental NEC and intestinal damage.

## Materials and Methods

### Isolation of AFSC-exos and HBM-exos

#### Isolation of AFSC-exos from hAFSCs Culture Supernatants

hAFSCs were a gift from the Stem Cell and Biomedical Material Key Laboratory of Jiangsu Province. hAFSCs were cultured with CHANG Amnio (Irvine) amniotic fluid cell culture medium containing 10% fetal bovine serum (FBS) (Hyclone) and 1% penicillin/streptomycin (Gibco) in a humidified incubator under 5% CO_2_ at 37 ℃. The cells were cultivated until they reached 60–80% confluence (24–48 h) in culture medium supplemented with Exosome-depleted FBS (Absin), and then the supernatant was collected by centrifugation for 5 min at 750 × *g*, 4 °C to deplete residual floating cells. The 60 ml supernatant was added into two 50 mL tubes and centrifuged again for 5 min at 1,500 × *g*, 4 °C to pellet larger cell debris. The supernatant was then centrifuged for 35 min at 14,000 × *g*, 4 °C to deplete residual micro vesicles. The supernatant was decanted into an ultracentrifugation tube (Beckman Coulter) and centrifuged for 2 h at 110,000 × *g*, 4 °C. The supernatant was discarded, the tubes were inverted on a paper towel, and left for 3–5 min until all remaining supernatant had soaked into the towel. The AFSC-exos pellet was resuspended in 1,000μL of phosphate-buffered saline (PBS) and strained through a sterile 0.22 μm disposable needle filter (Millipore). The concentration determined by BCA protein concentration Assay Kit (Beyotime) was 1.2 mg/ml. The AFSC-exos were stored at − 80 °C. They remained stable for several months, but repeated freeze–thaw-cycles should be avoided.

#### Isolation of HBM-exos from Human Breast Milk

Human breast milk was obtained from Affiliated Children's Hospital, Soochow University and was pooled from three different donors. 30 ml breast milk samples were collected into sterile tubes and immediately shipped on ice for processing in our laboratory. HBM-exos were purified using a series of ultracentrifugation steps as follows: 3000 × *g* for 15 min,12000 × *g* for 60 min, 35000 × *g* for 60 min, repeated three times to remove fat. The final supernatant was filtered through a sterile 0.22 μm disposable needle filter, followed by centrifugation 120,000 × *g* for 60 min, twice. The pellet was retained, resuspended in PBS, and strain through a sterile 0.22 μm disposable needle filter. The concentration determined by BCA protein concentration Assay Kit (Beyotime) was 1.5 mg/ml. The HBM-exos were stored at − 80 °C. They remained stable for several months, but repeated freeze–thaw-cycles should be avoided.

#### Characterization of AFSC-exos and HBM-exos

A Bradford Protein Concentration Determination Kit (Beyotime) was used to determine the concentrations of AFSC-exos and HBM-exos. Exosome morphologies were observed using an FEI Tecnai G2 spirit transmission electron microscope (TEM; FEI, Eindhoven, the Netherlands). Antibodies against CD63 (Rabbit Polyclonal Antibody, 25682–1-AP,1:1000, Proteintech, SI, China) and CD81 (Mouse Monoclonal Antibody, 66866–1-lg,1:1000, Proteintech, Wuhan, China) were used to analyze the protein levels in the exosomes using western blotting. The size distribution of the AFSC-exos and HBM-exos were measured using tunable resistive pulse sensing Tunable Resistive Pulse Sensing (TRPS) analysis by a Zetasizer Nano ZS90 (Malvern Panalytical, Malvern, UK).

### Animals and the NEC Model

#### Induction and Treatment of NEC Model

All animal experiments were approved by the Animal Care Committee at Affiliated Children's Hospital, Soochow University (no.2021CS188), and all methods were performed according to national guidelines and regulations. Mouse pups were randomly assigned to each of the experimental groups to eliminate potential within-litter effects,. Experiments were designed to include six mice per group in each of the independent experiments. In vivo experiments were performed independently three times.

C57BL/6 wild-type (WT) mice were originally purchased from Ling Chang Biological Technology Co., LTD. (Shanghai, China). Based on a published murine model of NEC [13], NEC was induced in neonatal C57BL/6 six mice from postnatal day 6–10. Pups were hand-fed hypercaloric low lactose formula milk (ESBILAC) every 3 h by gavage. In addition, these pups were stressed every 12 h using asphyxia (100% nitrogen gas for 60 s) followed 5 min later by cold stress (placement in a refrigerator at 4 °C for 5 min). The WT pups remained with their mothers to breastfeed. The NEC group were induced for NEC by hypertonic feeding, asphyxia and cold stress. The treatment group mice received a gavage of either AFSC-exos (5 μg) or HBM-exos (5 μg) on postnatal days 8–10, three times a day, during NEC induction. On postnatal day 10, pups were sacrificed, and their intestines (including duodenum, jejunum, ileum, ileocecal junction, and colon), peripheral blood, and brain were harvested for further studies.

#### Physiological Status, Weight, and Survival Time

During the experiment, the appearance, reactivity and activity of the mice were observed and photographed. Before and during the experiment, the weight of each pup was determined regularly and accurately to 0.01 g, the number of deaths and the survival time of each group of pups were also recorded.

### NEC- Associated Intestinal Injury Score and Crypt Counting based on Hematoxylin and Eosin (HE) Staining

#### Intestinal Injury Score

The entire small bowels were immediately fixed in 10% buffered formaldehyde for 3d. Afterwards, the samples were embedded in paraffin, sectioned at 4 µm thickness, and stained with HE using standard protocols. Intestinal injury scores were assigned by two investigators based on the histological injury scoring system described by Caplan et al. [14]: NEC scores from 0 to 4 were assigned for each sample. Grade 0: intact epithelium with villus structure; 1: superficial epithelial cell sloughing; 2: mid-villous necrosis; 3: complete villous necrosis; and 4: transmural necrosis. Samples with histological scores of 3 or higher were considered positive for NEC.

#### Ileum Crypt Count

The small intestinal gland, also known as the intestinal crypt, is formed by the subepithelial recess to the lamina propria of the villi root. The structure is that the subepithelial layer of the villi roots buries into the lamina propria to form a tubular small intestinal gland, or intestinal crypt. Therefore, the epithelium of the small intestinal gland and the villi are continuous, and the small intestinal gland directly opens into the intestinal lumen. As part of the small intestine, the ileum has a crypt structure called Ileum crypt. We selected four groups of mice to count the number of crypts in the cross section of ileum using HE staining.

### Immunohistochemistry (IHC)

For IHC of the mouse ileum and brain tissue, 4 μm sections were cut, deparaffinized, and stained. **Leucine rich repeat containing G protein-coupled receptor 5** (Lgr5) is a marker of the intestinal stem cells(ISCs),ISCs are the guardians of the intestine. Here, we measured Lrg5 to reflect the number of intestinal stem cells and evaluate the intestinal state. Immunohistochemistry staining for Lgr5and **Interleukin-6** (IL-6) in the ileum were performed to assess regeneration and inflammation. After blocking, tissue sections were incubated with primary antibodies overnight at 4 °C (primary antibodies recognizingLgr5(Rabbit Polyclonal Antibody, AF0165,1:400, Beyotime, Shanghai,

China) andIL-6(Rabbit Polyclonal Antibody, NBP2-16957,1:2000, Novus, Colorado, USA). Thereafter, a DAB Detection Kit (Beyotime) was used for secondary antibodies incubation and color development. Positively stained cells were counted in each tissue section. **Myelin basic protein** (MBP) is the main myelin protein in the central nervous system (CNS), which is a diagnostic index reflecting the substantial damage of the CNS, especially the demyelination. **Ionized calcium-binding adapter molecule 1 (Iba1)** is a specific marker in microglia in the central nervous system, which can quickly sense neurological disorders and be activated in response to brain lesions or injuries. Antibodies against MBP (Rabbit Polyclonal Antibody, AF7527, 1:200, Beyotime, Shanghai, China) and Iba1 (Rabbit Monoclonal Antibody, 17198S,1:800, Cell Signaling Technology, Massachusetts, USA) proteins were used to analyze the expression of each protein in brains using the same IHC method, to assess NEC- associated brain injury. IHC scoring was based on the intensity and distribution of positive DAB staining using a score of 0 (absent) to 3 (high intensity, widely distributed) and was evaluated by three blinded assessors.

### RNA-seq Analysis

Total RNA was extracted from the mouse ileum using an RNeasy Micro Kit (Qiagen Germany) following the manufacturer's protocol. Total RNA was delivered to The Shanghai Biotechnology Corporation (Shanghai, China) for sequencing with Illumina HiSeq2500.FPKM(fragments per kilobase of exon model per million mapped reads) was used to standardize the gene expression level. Statistics for differentially expressed genes(DEGs) were calculated by a *q* value ≤ 0.05 and fold change ≥ 2(RNA-seq:GSE143497). A heatmap was plotted using https://hiplot.com.cn/, a free online platform for data analysis and visualization.

### Gene Ontology (GO) and Kyoto Encyclopedia of Genes and Genomes (KEGG) Enrichment Analysis

For functional enrichment analysis, all DEGs were mapped to terms in the GO databases, and then significantly enriched GO terms were searched among the DEGs, using *p* < 0.05 as the threshold. GO term analysis was classified into three subgroups, namely biological process (BP), cellular component (CC) and molecular function (MF). All DEGs were mapped to the KEGG database, and searched for significantly enriched KEGG pathways at *p* < 0.05. Histogram, cnetplot, and bubble charts were plotted using https://www.bioinformatics.com.cn, a free online platform for data analysis and visualization.

### IEC-6 and IEC-18 Cell Lines

Rat IEC-6 and IEC-18 cells were obtained from the Shanghai Fuheng Biotechnology Co., LTD. (Shanghai, China). IEC-6 is crypt epithelial cells of rat small intestine.IEC-18 is Rat ileal epithelial cells. Cells were routinely cultured in Dulbecco's modified eagle medium with high glucose (DMEM)-containing 10% FBS (Hyclone) and 1% penicillin/streptomycin (Gibco) in a humidified incubator under 5% CO_2_ at 37 ℃. IEC-6 and IEC-18 cells were exposed to lipopolysaccharide (LPS, 50 μg/mL, Sigma) for 24 h to induce epithelial injury *in vitro*. AFSC-exos (10 μg/mL) or HBM-exos (10 μg/mL) were added separately to relieve inflammation of IECs and restore healing and repair abilities.

### Enzyme-Linked Immunosorbent Assay (ELISA)

Mice serum was obtained by centrifugation from peripheral blood and used for ELISA. In addition, IEC cell culture supernatants were collected for ELISA. According to the manufacturer’s protocol, the levels of IL-6 in the mouse serum and secreted IL-6 in IEC cell culture supernatants were determined using an ELISA kit (Mouse IL-6 ELISA Kit,EK206, MULTI SCIENCES; Rat IL-6 ELISA Kit, EK306, MULTI SCIENCES). Three times ELISA analyzes were repeated for each sample A Multiskan FC photometer (Thermo Scientific) was used to detect the absorbance of each sample at 450 nm.

### Quantitative Real-Time Reverse Transcription PCR (qRT-PCR)

Total RNA was isolated from IEC-6, IEC-18 and mouse ileum by using an RNeasy Micro Kit (Qiagen Germany) and reverse transcribed into cDNA a PrimeScript Reverse Transcriptase kit (Takara). Next, the cDNA was used as the template for the quantitative real-time PCR (qPCR) step of the qRT-PCR protocol, performed using a HiScript II One Step qRT-PCR SYBR Green kit (Vazyme) on an iQ5 Real-time PCR Detection system (Bio-Rad,). The relative gene expression was analyzed using the 2^–ΔΔCT^ method [15]. β-actin was used as reference gene. The primers for Mouse *β-actin*, Mouse ***Lgr5***, Mouse *Il6*, Rat *β-actin* and Rat *Il6* are listed in Table S1. Finally, we detected Mouse β-actin, Lgr5, Il6 in mouse ileum, and Rat β-actin and Il6 in IEC-6 and IEC-18.

### Wound-Healing Assay

IECs were divided into four groups: Control, LPS-induced, LPS-induced + AFSC-exos, and LPS-induced + HBM-exos. After the IEC culture reached 90–100% confluence, a scratch was made in the cultured monolayer. Then, the cells were washed with PBS to remove detached cells. Images of the wounded area were captured at different time points (0, 12, and 24 h; or 0, 24, and 48 h) under a microscope (Nikon Eclipse Ti, Tokyo, Japan) connected to a Nikon camera using NIS Elements software (Nikon).

### Migration Assay

A Transwell Permeable Support (Corning) was used for the migration assay. Coculture experiments were performed by seeding IECs (2 × 10^4^) in the upper chamber and culturing them with 200μL of four different media: Serum-free DMEM, Serum-free DMEM + LPS, Serum-free DMEM  + LPS + AFSC-exos or Serum-free DMEM + LPS + HBM-exos; 600μL of DMEM containing 10% FBS was placed in the lower chamber of the system. After incubation for 24 h, the migrated cells were stained with crystal violet and counted.

### Statistical Analysis

All experiments were conducted by investigators blinded to the group allocation during the experiment and/or when assessing the outcome. No data were excluded from the analysis. GraphPad Prism software (GraphPad) was used to draw diagrams and perform the following statistical analysis. All the results passed the normal distribution test (Kolmogorov–Smirnov test) and are presented as means ± SD. Two groups were compared by unpaired Student’s t test, while more than two groups were assessed through one-way analysis of variance followed by Bonferroni test. A p value < 0.05 was considered significant.

## Results

### Comparison of AFSC-exos and HBM-exos for the Treatment of NEC

**Fig. 1:**
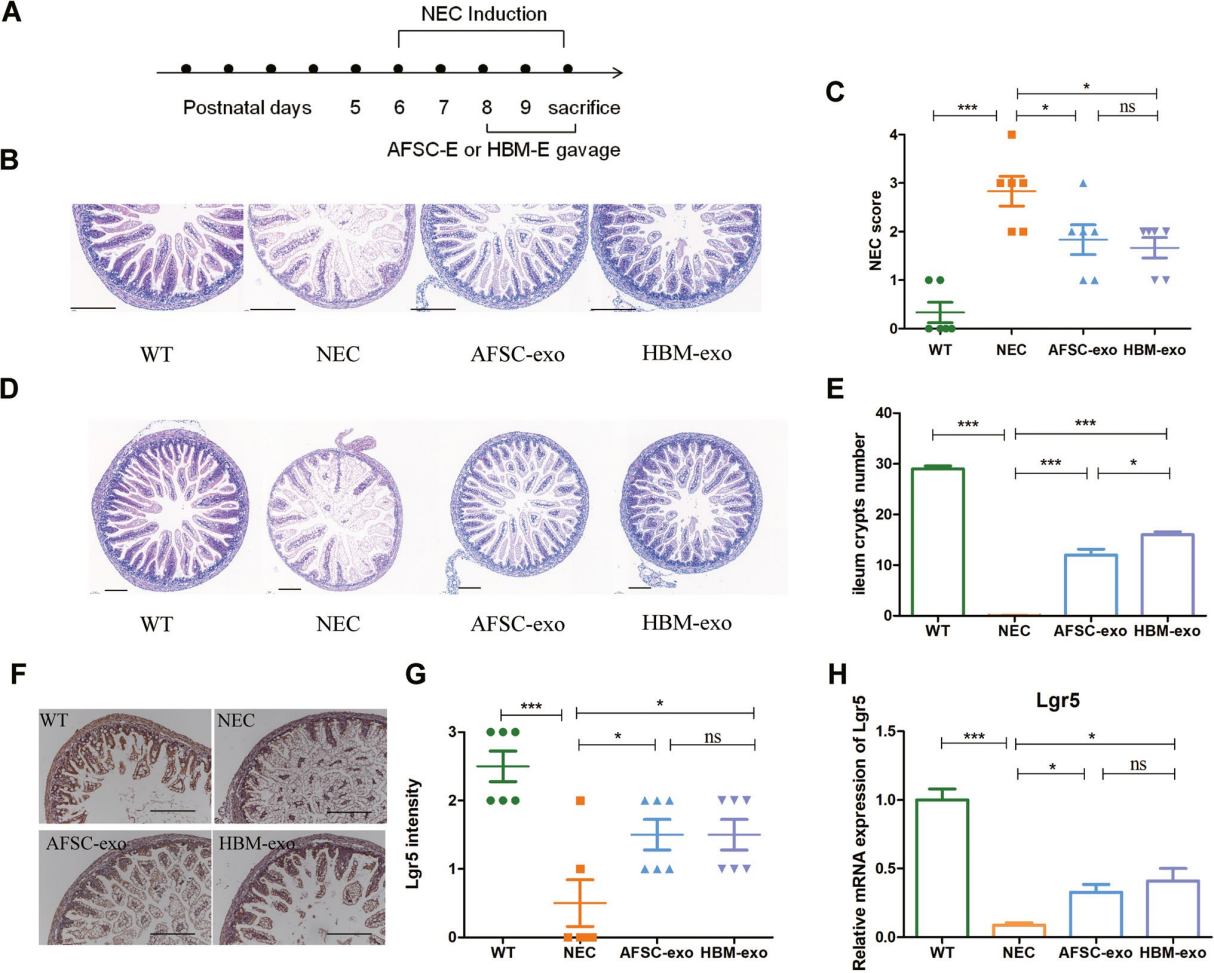
AFSC-exos and HBM-exos alleviates intestinal injury in NEC model mice. (**A**) NEC induction was conducted on postnatal (p) days 6–10, with intragastric administration of AFSC-exos and HBM-exos given on p8–10. (**B**) Histopathology of ileal sections from mice administered with AFSC-exos or HBM-exos during NEC induction showed less intestinal histological injury compared with mice in NEC group. Scale bars: 100 µm. (**C**) Administration of AFSC-exos or HBM-exos during NEC significantly decreased the intestinal histological scores. (**D**) Administration of AFSC-exos and HBM-exos restored reduced ileum crypts compared with those in NEC mice, as shown by the histopathology of ileal sections. Scale bars: 100 µm. (**E**) Number of ileum crypts in ileum sections of the mice in each group. (**F, G**) Immunohistochemical assessment of Lgr5 in the ileum from the WT (*n* = 6), NEC (*n* = 6), AFSC-exos (*n* = 6), and HBM-exos (*n* = 6) groups. Scale bars: 100 µm. Scatter plot indicating the means of individual signal intensity values, which are shown as dots. Data are presented as means ± SD. *P < 0.05, **P < 0.01 and ***P < 0.001; using an unpaired t test or one-way ANOVA with post-hoc tests, as appropriate. (**H**) Administration of AFSC-exos or HBM-exos during NEC increased the gene expression of *Lgr5*, an ISCs marker, compared with that in NEC mice

Although both AFSC-exos and HBM-exos have certain intervention effects on NEC models [16, 17], the specific molecular mechanisms need to be further investigated. In view of this, we successfully constructed a NEC mouse model (Fig. 1A) and used the isolated AFSC-exos and HBM-exos to intervene (Fig. S1.The full length uncropped original western blots involved were shown in Fig. S6). The mice in the NEC group had different degrees of intestinal injury compared with the WT group, especially the ileum (Fig. S2). However, after treatment with AFSC-exos and HBM-exos, NEC- associated intestinal injury was alleviated and the NEC score was significantly decreased (Fig. 1B, C). Interestingly, the ileal crypts number was more significantly recovered after HBM-exos intervention treatment compared with AFSC-exos (Fig. 1D, E).

The intestinal stem cells(ISCs) are the guardians of the intestine, maintaining intestinal homeostasis and function at all times [18]. Thus, we monitored the expression of Lgr5^+^ intestinal stem cells in intestinal tissues of each experimental group [19], and found that the number of Lgr5^+^ intestinal stem cells in the NEC group was sharply reduced compared with the WT group (Fig. 1F, G), but after the intervention treatment with AFSC-exos and HBM- exos, the number of Lgr5^+^ intestinal stem cells increased significantly (Fig. 1F, G). Similarly, the level of Lgr5 gene expression is reduced in the intestine of NEC (Fig. 1H), but after the intervention treatment with AFSC-exos and HBM- exos, the level of Lgr5 gene expression increased significantly (Fig. 1H). Although both AFSC- exos and HBM- exos had therapeutic effects on NEC mice, this short-term intervention did not result in significant improvements in individual size, weight, or survival cycle (Fig. S3). These findings suggest that both AFSC- exos and HBM- exos have therapeutic effects on NEC mice, but the specific intervention mechanisms involved are different.

### Both HBM-exos and AFSC-exos have Inhibitory Effects on Inflammation *In Vivo*

**Fig. 2:**
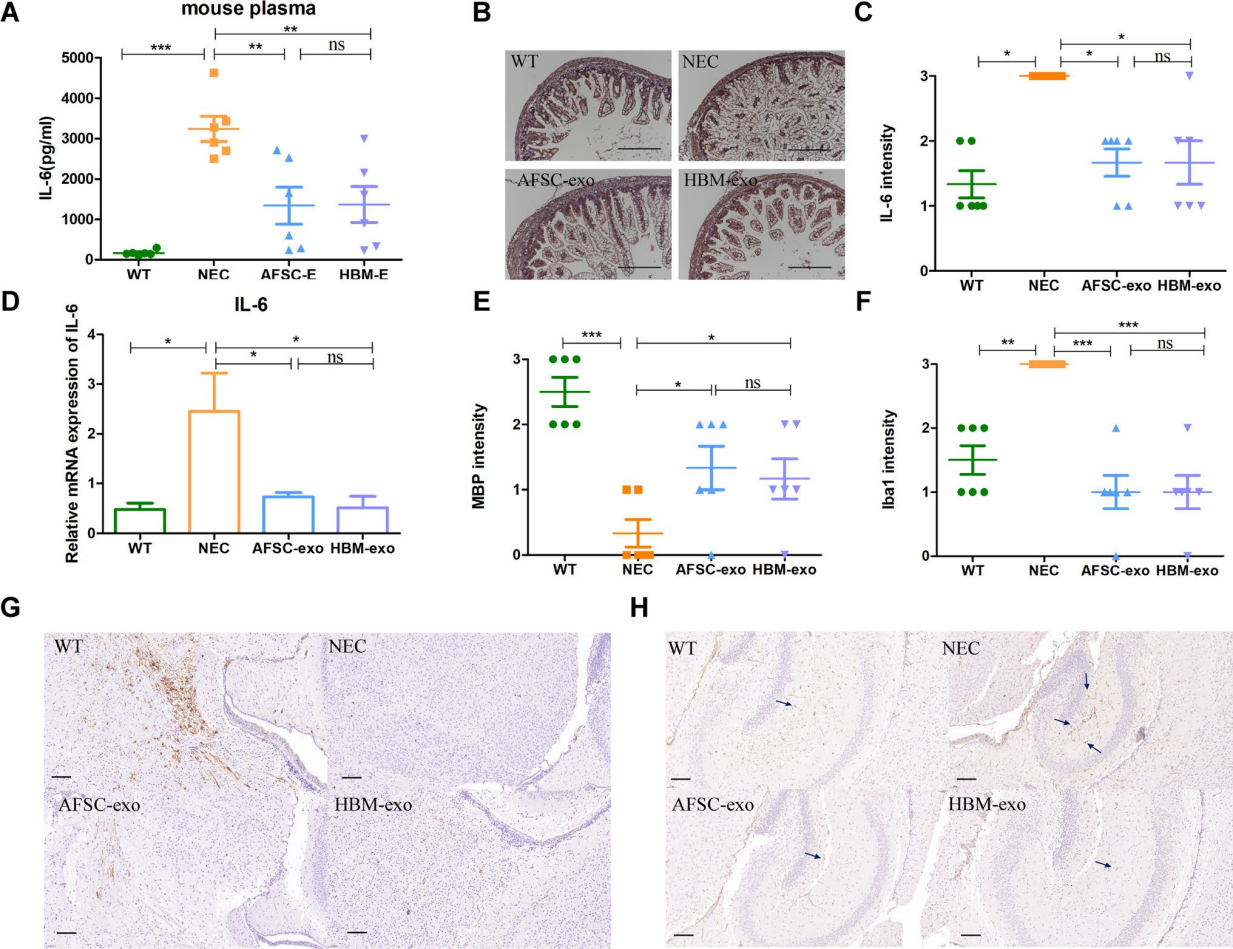
AFSC-exos and HBM-exos attenuated inflammation and NEC- associated brain injury. (**A**) Levels of IL-6 in peripheral blood serum from mice administered with AFSC-exos and HBM-exos were reduced compared with those in NEC mice. (**B, C**) Immunohistochemical assessment of IL-6 in the ileum from the WT (*n* = 6), NEC (*n* = 6), AFSC-exos (*n* = 6), and HBM-exos (*n* = 6) groups. Scale bars: 100 µm. Scatter plot indicating the means of individual signal intensity values, which are shown as dots. Data are presented as means ± SD. *P < 0.05, **P < 0.01 and ***P < 0.001; using an unpaired t test or one-way ANOVA with post-hoc tests as appropriate. (**D**) Administration of AFSC-exos or HBM-exos during NEC had decreased gene expression of *Il6*, a pro-inflammatory cytokine, in the ileum compared with that in NEC mice. Mice with NEC developed marked brain injury, manifested as a loss of myelin basic protein (MBP) (**E, G**) and an increase of Iba1 (**F, H**) in the midbrain, which was recovered after gavage of AFSC-exos and HBM-exos. Scale bars: 100 µm. Scatter plot indicating the means of individual signal intensity values, which are shown as dots. Data are presented as means ± SD. *P < 0.05, **P < 0.01 and ***P < 0.001; using an unpaired t test or one-way ANOVA with post-hoc tests as appropriate

The alleviation of the inflammatory response is also one of the important indicators for the assessment of the effect after NEC treatment. In the exosomes-treated study group, AFSC-exos and HBM-exos reduced the systemic pro-inflammatory cytokine IL-6 (Fig. 2A) and intestinal (Fig. 2B, C) protein and mRNA expression (Fig. 2D) of the pro-inflammatory cytokine IL-6. Here, we found that the treatment of NEC model mice with AFSC-exos and HBM-exos intervention not only resulted in the alleviation of their systemic response (Fig. 2A), but also improved the local intestinal inflammatory microenvironment (Fig. 2B-D).

In addition, we also monitored pathological changes in the brains of the mice in each experimental group, since brain disease is also one of the complications of NEC. As reported in the literature [20], the brain tissue of NEC model mice showed pathological changes with a significant decrease in the expression level of Myelin basic protein (MBP) protein in the midbrain region (Fig. 2E, G). MBP plays an important role in maintaining the stability of the structure and function of the myelin sheath of the central nervous system, and is a key indicator to judge the severity of nerve injury. In addition, microglia were aberrantly activated in localized areas of brain tissue in NEC model mice (Fig. 2F, H), which generally showed a dramatic increase in number and aberrant activation in response to increased neurological disorders or inflammatory responses. Interestingly, both AFSC-exos intervention and HBM-exos intervention can well ameliorate the pathological damage of brain tissue, mainly by upregulating the expression of MBP and inhibiting the abnormal proliferation of microglia (Fig. 2E-H). The above results demonstrated that AFSC-exos and HBM-exos decreased systemic and ileum inflammation during experimental NEC, and reduced NEC- associated brain injury.

### HBM-exos tend to Suppress the Inflammatory Response, but AFSC-exos Tend to Regulate Migration of Intestinal Epithelial Cells *In Vitro*

**Fig. 3:**
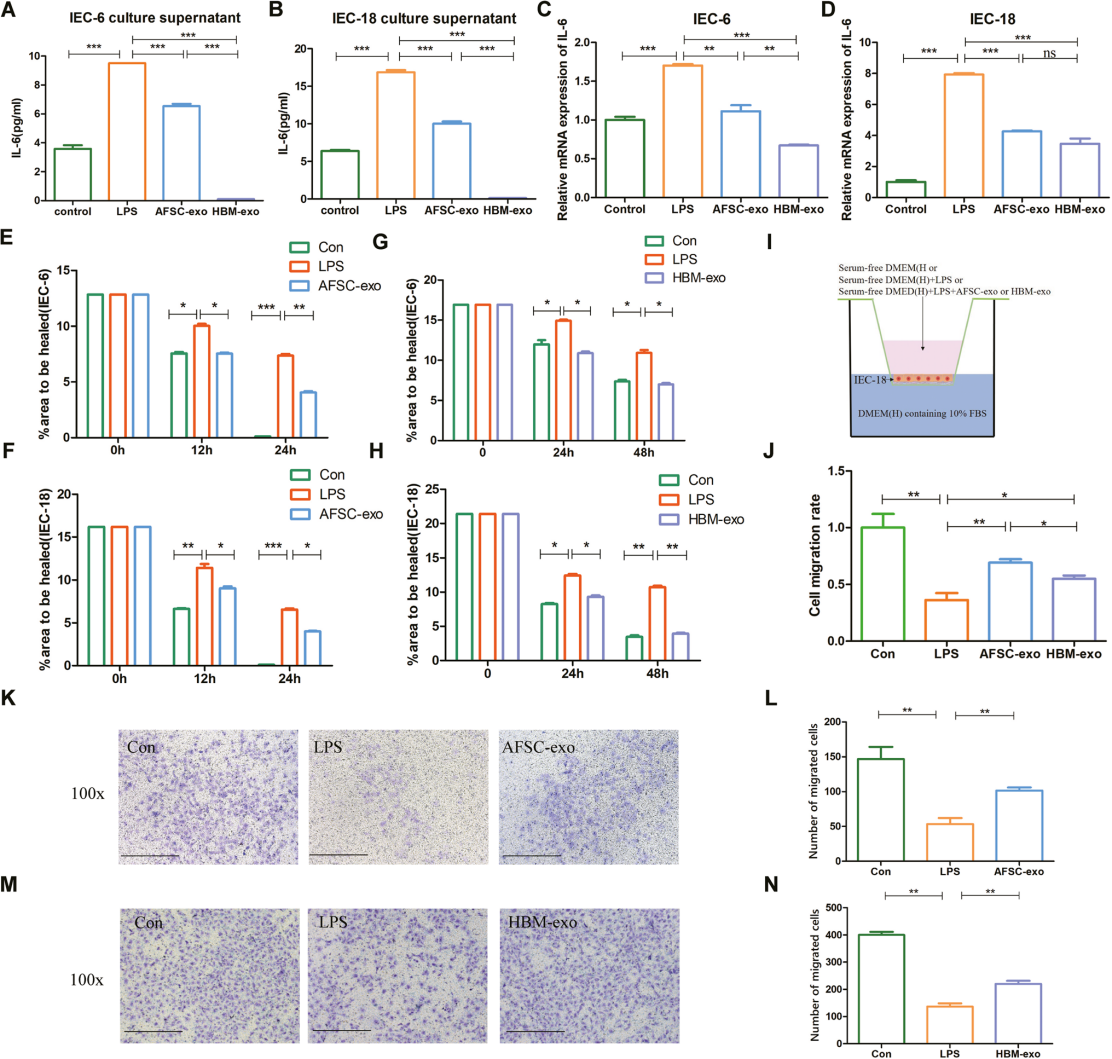
AFSC-exos and HBM-exos decreased IEC inflammation and restored their healing and repair ability in vitro. (**A**) AFSC-exos and HBM-exos decreased soluble-IL-6 secreted by LPS-induced injured IEC-6. (**B**) AFSC-exos and HBM-exos decreased soluble-IL-6 secreted by LPS-induced injured IEC-18. (**C**) AFSC-exos and HBM-exos reduced *Il6* expression in IEC-6 (**D**) AFSC-exos and HBM-exos reduced *Il6* expression in IEC-18. (**E, F**) Light microscopy images of the wound-healing area in cultures of IEC-18 cells, showing that their healing ability markedly improved after using AFSC-exos. (**G, H**) Light microscopy images of the wound-healing area in cultures of IEC-18 cells, showing that their healing ability were markedly improved after using HBM-exos. (**I**) Transwell system was used to verify the migration ability of IEC-18 cells. (**J**) The cell migration rate was normalized to the control group. (**K, L**) AFSC-exos increased IEC-18 cell migration. (**M, N**) HBM-exos increased IEC-18 cell migration. Scale bars:50 µm

Functional changes in epithelial cells are also important in the assessment of the efficacy of NEC interventions, with IEC being a long-standing model for studying NEC-related mechanisms at the cellular level. There was an increase in the secretion of inflammatory marker IL-6 compared with that in the control when IEC-6 (Fig. 3A) and IEC18 (Fig. 3B) cells were exposed to LPS to induce injury, and both AFSC-exos and HBM-exos could decrease the level of soluble-IL-6 secreted by injured IECs (Fig. 3A, B). In addition, both types of exosomes reduced *Il6* gene expression in IEC-6 (Fig. 3C) and IEC-18 cells (Fig. 3D). Both AFSC-exos and HBM-exos significantly inhibited LPS-induced upregulation of the inflammatory factor IL-6 in intestinal epithelial cells (IEC-6, IEC-18) (Fig. 3A-D). In particular, HBM-exos better inhibited the protein and mRNA levels of IL-6 in LPS-stimulated IEC-6 cells (Fig. 3A-C). When the intestinal epithelium is impaired, the surrounding epithelial cells migrate to the site of injury and promote wound healing. Furthermore, we found that LPS stimulation resulted in diminished scratch healing in IEC-6 (Fig. 3E, G, Fig. S4A, Fig. S4C) and IEC-18 (Fig. 3F, H, Fig. S4B, Fig. S4D), but both AFSC-exos (Fig. 3E, F, Fig. S4A, Fig. S4B) and HBM-exos (Fig. 3G, H, Fig. S4C, Fig. S4D) intervention treatments could reverse the LPS effect to promote scratch healing (Fig. 3E-H; Fig. S4A-S4D). Interestingly, scratch healing in the AFSC-exos intervention group healed almost 24 h (Fig. 3E, F), while the HBM-exos intervention group required 48 h (Fig. 3G, H). We used the Transwell system to verify the migration ability of IEC-18 cells cocultured with or without AFSC-exos or HBM-exos (Fig. 3I). LPS caused a significant decrease in the ability of IEC-18 cells to migrate (Fig. 3K-N), whereas both AFSC-exos (Fig. 3K, L) and HBM-exos (Fig. 3M, N) increased IEC migration (Fig. 3J). Similarly, the reversal effect of AFSC-exos intervention in diminishing epithelial cell migration capacity caused by LPS was most pronounced (Fig. 3I-N). Collectively, these *in vitro* findings suggested that that HBM-exos preferred to suppress the inflammatory response, while AFSC-exos preferred to regulate migration of intestinal epithelial cells.

### Bioinformatic Analysis Reveals Different Molecular Mechanisms of AFSC-exos and HBM-exos Intervention Therapy

We have demonstrated that both AFSC-exos and HBM-exos have therapeutic effects on NEC mice, but they must be involved in the damage repair process of NEC through different regulatory approaches. In view of this, we collected ileal tissues from the above four experimental groups for transcriptome level sequencing, and then combined with bioinformatics analysis to reveal the different mechanisms of tissue damage repair between the two types of exosomes.

To ensure the accuracy of subsequent data used to mine the therapeutic mechanism, we first identified the upregulated differentially expressed genes (DEGs, *n* = 993) in the NEC group compared with the WT group, and then identified the downregulated DEGs (*n* = 20) after AFSC-exos treatment among these genes. At the same time, among the downregulated DEGs (*n* = 3625) in NEC group compared with the WT group, the upregulated DEGs (*n* = 86) after AFSC-exos treatment were identified. Similarly, among the upregulated DEGs (*n* = 993) in the NEC group compared with the WT group, 54 downregulated DEGs were identified after HBM-exos treatment. Among the 3625 downregulated DEGs in the NEC group compared with the WT group, 186 upregulated DEGs were identified after HBM-exos treatment. Here, we refer to these genes as post-treatment callback DEGs (Fig. S5). Subsequent enrichment analyses were all based on these post-treatment callback DEGs.

**Fig. 4:**
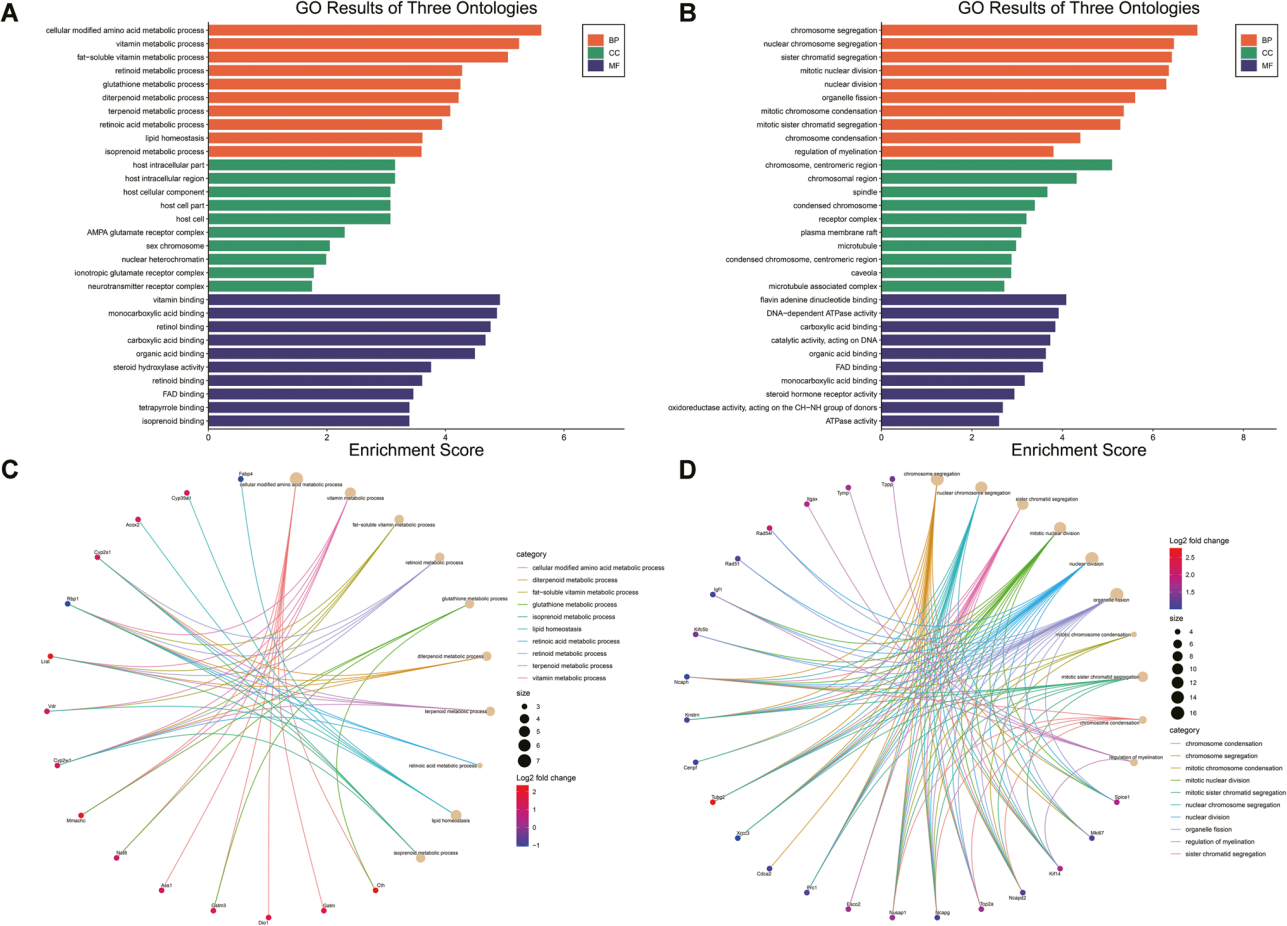
Significantly enriched Gene Ontology (GO) terms between ileum tissues from exosome-treated NEC mice and NEC model mice based on their functions. The top 10 biological process (BP) terms, cellular component (CC) terms, and molecular function (MF) terms in the enrichment analysis between ileum tissues from AFSC-exos-treated NEC mice and NEC model mice (**A**) or between HBM-exos-treated NEC mice and NEC model mice (**B**). The top 10 GO-BP terms based on differentially expressed genes (DEGs) in the centplot enrichment analysis between ileum tissues from AFSC-exos-treated NEC mice and NEC model mice **(C)** or between HBM-exos-treated NEC mice and NEC model mice (**D**). The sizes of the points in the figure represent the number of annotated genes in the biological process. The colors of the lines in the figure represent the different categories of biological process. The colors of the points in the figure represent the fold change of the annotated genes

Subsequently, we performed GO analysis of the enriched DEGs. The genes that changed significantly in ileal tissues before and after AFSC-exos treatment were mainly related to metabolic processes such as amino acids, vitamins and lipids, while the genes that changed significantly in ileal tissues before and after HBM-exos treatment were mainly related to functional changes such as chromosome segregation and condensation. In terms of cellular composition, AFSC-exos was more associated with intracellular components of the host cells, while AFSC-exos was more associated with the chromosomal mitotic region. In terms of molecular function, the two functions are also very different, with AFSC-exosome mainly associated with vitamin binding, while HBM-exos is associated with flavin adenine dinucleotide binding (Fig. 4A, B). Together, AFSC-exos and HBM- exos do mediate different biological processes involved in tissue damage repair (Fig. 4C, D).

**Fig. 5:**
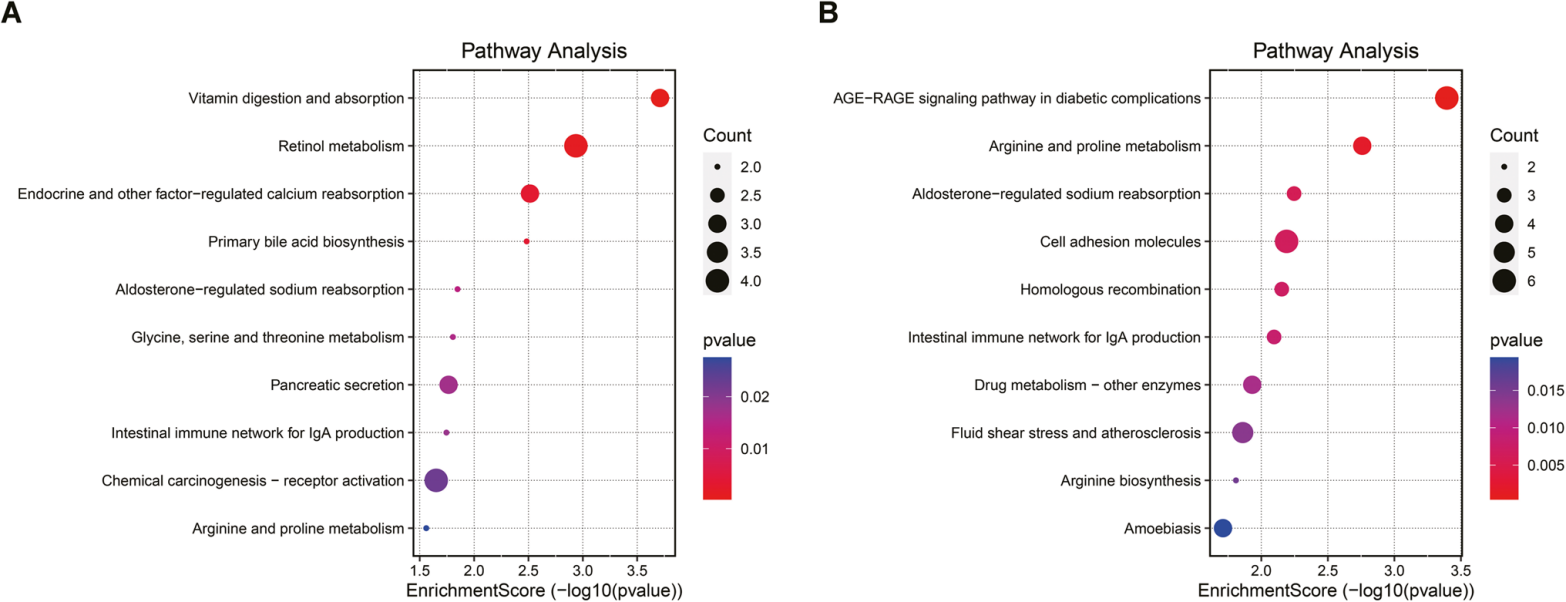
The top 10 enriched Kyoto Encyclopedia of Genes and Genomes (KEGG) pathway terms of differentially expressed genes (DEGs) for AFSC-exos intervention group(**A**)and HBM-exos intervention group(**B**). The size of the points in the figure represents the number of annotated genes in the functional pathway, the color of the points in the figure represents the -log10 (p-value), which represents the enrichment significance of the functional pathway, and the enrichment score is the score used to evaluate the enrichment degree of the functional gene/pathways

In addition, KEGG pathway enrichment analysis was conducted to explore the most significantly enriched pathways for the post treatment callback DEGs to better understand their biological functions in the treatment of NEC by AFSC-exos or HBM-exos. As a result, the top 10 KEGG enriched signaling pathways for the two intervention groups were obtained (Fig. 5A, B; Table S2, S3). The AFSC-exos group is mainly involved in signaling pathways related to vitamin digestion and absorption and retinol metabolism, while the HBM-exos group is mainly involved in signaling pathways related to amino acid metabolism and aldosterone reabsorption. In conclusion, AFSC-exos and HBM-exos are involved in tissue damage repair through the regulation of their respective unique signaling pathways.

## Discussion

Although exosomes from AFSC and HBM can protect against NEC, their mechanisms remain unclear. Here, we compared the intervention effects of two types of exosomes on NEC model. Intragastric administration of two types of exosomes could improve experimentally NEC- induced intestinal injury, restored intestinal regeneration, inhibited inflammation, and NEC-related complications. Furthermore, they could reduce the inflammatory response of IECs, and restore migration ability. Mechanistically, RNA-Seq and GO/KEGG analyses revealed their different underlying mechanisms in protecting against NEC. Both bioinformatic findings and laboratory findings suggested that the exosomes from AFSC and HBM both have protective effects on NEC. However, our experimental studies mainly focus on the intestinal epithelium, while sequencing studies the whole intestinal tissue. In addition to epithelial cells, many kinds of cells are involved in bioinformatic findings, which provides directions for our future research.

Previous research has demonstrated that different types of stem cells (SCs) and HBM have the ability to reduce the incidence and severity of experimental NEC [3, 4, 21]. Amniotic fluid was first found to be effective against NEC when microinjected into the fetal intestine, where it reduced the severity of NEC in mouse models, possibly because AFSCs enhanced the healing of NEC [22, 23]. In further studies, intraperitoneal injection of AFSCs improved intestinal injury and function, inhibited intestinal inflammation, and promoted intestinal epithelial cell proliferation in NEC rats [22]. The therapeutic effects by AFSCs are primarily achieved in a paracrine manner [22]. Exosomes, as important effector molecules of paracrine action, have been proven to play biological roles in many diseases [8–10].

Several stem cell-derived exosomes have been evaluated for NEC treatment [24]. However, current studies have been limited to the therapeutic effects on NEC- associated intestinal injury and inflammation. NEC is not only an intestinal disease [25, 26], but its broader sequelae include systemic inflammation, hypoxia, ischemia, and multi-system organ dysfunction in severe cases, particularly affecting brain function [27]. The alleviating effect of AFSC-exos and HBM-exos on systemic inflammation in our study has been mentioned previously. At present, NEC- associated brain injury is characterized by microglial activation, evidenced by increased expression of Iba1, accompanied by white matter loss, manifested by a loss of MBP [20]. Here, our results revealed that AFSC-exos and HBM-exos protected against NEC- associated brain injury by recovering the expression of MBP and Iba1.

As mentioned before, exosomes are not only derived from stem cells, but also are present in body fluids, for instance, HBM. Breast milk is the only factor consistently shown to improve NEC [28, 29]. Our previous study demonstrated that HBM-exos affect NEC by maintaining the integrity of the NEC-disrupted intestinal epithelial barrier [12]. In this study, we further verified the therapeutic effect of HBM-exos on experimental NEC by rescuing intestinal injury, restoring epithelial regeneration, and inhibiting intestinal inflammation. Moreover, it has been confirmed that HBM-exos could protect villus integrity from injury and restore cell proliferation [30]. These results are consistent with our results. In fact, early human breast milk, such as colostrum, contains more exosomes than mature breast milk [31]. Researchers have attempted to compare the effects of different stages of breast milk on NEC treatment. In an *in vitro* model [32], HBM-exos from colostrum had the best ability to inhibit proinflammatory reactions, which indicated that colostrum was the best source of HBM-exos. Colostrum is scarce and precious; therefore, it is difficult to collect in significant quantities. In the future, if there is an opportunity to collect breast milk at different stages, we could explore and compare the therapeutic effect of colostrum, transition, and maturation on NEC-associated broader sequelae, including systemic inflammation, hypoxia, ischemia, and multi-system organ dysfunction that are triggered in severe cases.

AFSC-exos were identified to improve NEC-disturbed intestinal regeneration through Wnt signaling [33]. The mechanism by which HBM-exos reduce the incidence of NEC is partly due to the activation of the Wnt/β-catenin signaling pathway, which increases intestinal stem cells activity and protects cells from oxidative stress [34]. Our results also confirmed both AFSC-exos and HBM-exos restored epithelial regeneration, as evidenced by the number of ileum crypts and increased Lgr5 expression in ISCs in experimental NEC. Besides IECs, there are many kinds of intestinal cells, such as Goblet cells, Paneth cells, intestinal stem cells, and even immune cells which all play a role in the pathogenesis of NEC [35]. Therefore, the mechanistic study should not be limited to the study of epithelial cell regeneration function in the future research.

Then we used bioinformatic analysis to explore the therapeutic mechanism of AFSC-exos and HBM-exos, respectively. The enrichment analysis of GO biological process showed that intestinal amino acids, vitamins, retinol metabolism, and lipid homeostasis were out of balance in mice with NEC. Previous studies have also shown that premature infants demonstrate metabolic differences at birth. Metabolite abnormalities progress in parallel to significant differences in nutritional delivery, signifying metabolic dysfunction in premature newborns prior to NEC onset [36]. Our GO enrichment found that these metabolic pathways and homeostasis were corrected when AFSC-exos were administered. In addition to regulating nutritional metabolism and homeostasis in mice, consistent with the above GO analysis, KEGG enrichment results showed that AFSC-exos administration also corrected calcium (Ca) and sodium (Na) reabsorption and restored the intestinal expression of immunoglobulin A (IgA). Together, AFSC-exos could simultaneously act on NEC through multiple mechanisms. Therefore, future studies on the potential mechanism of AFSC-exos in the treatment of NEC should focus more on the simultaneous regulation of nutrition and maintenance of intestinal immune network homeostasis.

Unlike the underlying mechanism by which AFSC-exos treat NEC, GO-BP enrichment analysis showed that genes related chromosome segregation, mitotic nuclear division related, organelle fission, and regulation of myelination were significantly upregulated during HBM-exos treatment of NEC. These results indicated that HBM-exos were more focused on regulating biological processes of growth and development. It has been documented that genetic variation in chromosomes is associated with an increased risk of NEC [37, 38]. Our results and those of previous studies indicate that HBM-exos have good prospects for NEC caused by genetic metabolism or abnormal growth and development. KEGG pathway analysis of the DEGs highlighted the importance of cell adhesion molecules, homologous recombination, and advanced glycation during the process of HBM-exos administration.

In conclusion, the above results suggest that both AFSC-exos and HBM-exos can be used for the interventional treatment of NEC. In particular, both are involved in intestinal injury repair through different regulatory approaches. Moreover, some key mechanisms of AFSC-exos and HBM-exos involved in NEC injury repair were elucidated by RNA seq combined with bioinformatics, thus facilitating the exploration of new targets for NEC intervention therapy.

## Supplementary Information

Below is the link to the electronic supplementary material.

Supplementary file1 (DOCX 22 KB)

ESM 1 (PNG 970 kb)

High resolution image (TIF 18025 kb)

ESM 2 (PNG 4417 kb)

High resolution image (TIF 26279 kb)

ESM 3 (PNG 567 kb)

High resolution image (TIF 16324 kb)

ESM 4 (PNG 3607 kb)

High resolution image (TIF 38156 kb)

ESM 5 (PNG 1326 kb)

High resolution image (TIF 60828 kb)

ESM 6 (PNG 864 kb)

High resolution image (TIF 15183 kb)

## Data Availability

The corresponding author will provide the original data used to support the findings of this study upon reasonable request.
