# Supplementary material for: Comparison and Investigation of Exosomes from Human Amniotic Fluid Stem Cells and Human Breast Milk in Alleviating Neonatal Necrotizing Enterocolitis
Source: Stem Cell Rev Rep. 2022 Nov 16;19(3):754–66. doi: 10.1007/s12015-022-10470-5 (PMC10070207; doi:10.1007/s12015-022-10470-5)
Supplement: Supplementary file 1 — Supplementary file1 (DOCX 22 KB) [file 12015_2022_10470_MOESM1_ESM.docx]

1. **Supplementary Figure legends**

**Figure S1. Characterization of** **AFSC-exos and HBM-exos. (A)** TEM of AFSC-exos and HBM-exos. **(B)** AFSC-Exos and HBM-Exos expressed exosomal markers CD63 and CD81 **(C)** Sizes of the majority of the AFSC-exos and HBM-exos. Scale bars: 100 nm.

**Figure S2. The NEC mouse model was successfully induced. (A)** Representative histopathology sections of the duodenum, jejunum, ileum, ileocecal junction, and colon sections from WT and NEC mice. **(B)** NEC mice had increased intestinal injury histological scores for the above intestinal segments. Scale bars: 100 µm.

**Figure S3. The physiological status of the mice in each group. (A)** Morphological appearance. **(B)** Weight. **(C)** Survival curves.

**Figure S4. AFSC-exos and HBM-exos restored the healing ability of LPS-induced injured IEC-6 cells *in vitro*.** **(A, B)** Light microscopy images of the wound-healing area in cultures of IEC-6**(A)** and IEC-18**(B)**, showing that their healing ability markedly improved after using AFSC-exos. **(C, D)** Light microscopy images of the wound-healing area in cultures of IEC-6**(C)** and IEC-18**(D)**, showing that their healing ability was markedly improved after using HBM-exos. Scale bars: 100 µm.

**Figure S5.** **A heatmap** **of post-treatment callback DEGs.**

**Figure S6. Full length uncropped original western blot of CD63(A) and CD81(B).**

1. **Supplementary tables**

**Table S1.** **qRT-PCR primers**

| **Gene** | **Forward primer (5’- 3’)** | **Reverse primer (5’- 3’)** |
| --- | --- | --- |
| Mouse *β-actin* | GTGACGTTGACATCCGTAAAGA | GCCGGACTCATCGTACTCC |
| Mouse *Lgr5* | CCTACTCGAAGACTTACCCAGT | GCATTGGGGTGAATGATAGCA |
| Mouse *Il6* | CTGCAAGAGACTTCCATCCAG | AGTGGTATAGACAGGTCTGTTGG |
| Rat *β-actin* | AGGCCAACCGTGAAAAGATG | ACCAGAGGCATACAGGGACAA |
| Rat *Il6* | ATATGTTCTCAGGGAGATCTTGGAA | GTGCATCATCGCTGTTCATACA |

**Table S2.** **Top 10 KEGG enriched pathways and related genes after AFSC-exos administration**

| **ID** | **Description** | | | | **geneID** |
| --- | --- | --- | --- | --- | --- |
| mmu04977 | | Vitamin digestion and absorption | | | Slc23a1/Lrat/Mmachc |
| mmu00830 | | Retinol metabolism | Cyp2w1/Cyp3a25/Lrat/Cyp2s1 | | |
| mmu04961 | | Endocrine and other factor-regulated calcium reabsorption | | Klk1/Vdr/Atp1a2 | |
| mmu00120 | | Primary bile acid biosynthesis | | | Acox2/Cyp39a1 |
| mmu04960 | | Aldosterone-regulated sodium reabsorption | | | Atp1a2/Hsd11b2 |
| mmu00260 | | Glycine, serine and threonine metabolism | | | Cth/Gatm |
| mmu04972 | | Pancreatic secretion | | | Cpb1/Atp1a2/Cckar |
| mmu04672 | | Intestinal immune network for IgA production | | | Ccl28/Pigr |
| mmu05207 | | Chemical carcinogenesis - receptor activation | | | Cyp3a25/Vdr/Gstm3/Cdc6 |
| mmu00330 | | Arginine and proline metabolism | | | Prodh/Gatm |

**Table S3.** **Top 10 KEGG enriched pathways and related genes after HBM-exos administration**

| **ID** | **Description** | | | | **geneID** | | |
| --- | --- | --- | --- | --- | --- | --- | --- |
| mmu04933 | | AGE-RAGE signaling pathway in diabetic complications | | Nox4/Col4a3/Col4a5/Egr1/Sele/Ccl2 | | | |
| mmu00330 | | Arginine and proline metabolism | | | Aoc1/Prodh/Cndp1/Prodh2 | | |
| mmu04960 | | Aldosterone-regulated sodium reabsorption | | | | Atp1a2/Igf1/Hsd11b2 | |
| mmu04514 | | Cell adhesion molecules | Itgam/H2-Eb1/Cldn15/Cadm3/  Sele/H2-Q10 | | | | |
| mmu03440 | | Homologous recombination | | | Rad51/Xrcc3/Rad54l | | |
| mmu04672 | | Intestinal immune network for IgA production | | | | | H2-Eb1/Ccl28/Pigr |
| mmu00983 | | Drug metabolism - other enzymes | | | Tymp/Gsta3/Gstm3/Tk1 | | |
| mmu05418 | | Fluid shear stress and atherosclerosis | | | Gsta3/Gstm3/Ass1/Sele/Ccl2 | | |
| mmu00220 | | Arginine biosynthesis | | | Cps1/Ass1 | | |
| mmu05146 | | Amoebiasis | | | Itgam/Col4a3/Col4a5/Cxcl1 | | |
