# Supplementary figures and images for: Comparison and Investigation of Exosomes from Human Amniotic Fluid Stem Cells and Human Breast Milk in Alleviating Neonatal Necrotizing Enterocolitis
Source: Stem Cell Rev Rep. 2022 Nov 16;19(3):754–66. doi: 10.1007/s12015-022-10470-5 (PMC10070207; doi:10.1007/s12015-022-10470-5)

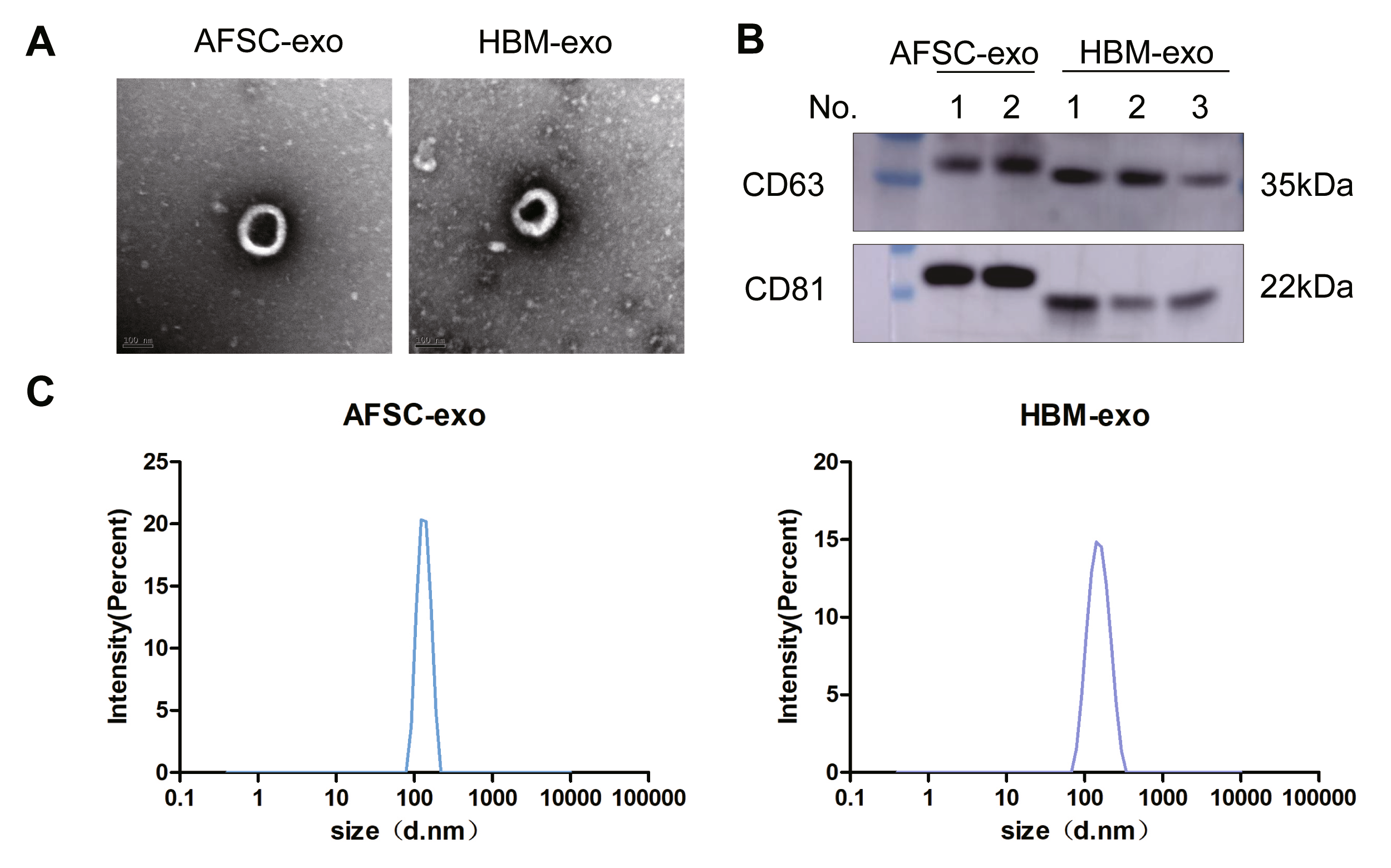

Supplement: Supplementary file 2 — (PNG 970 kb) [file 12015_2022_10470_Fig6_ESM.png]

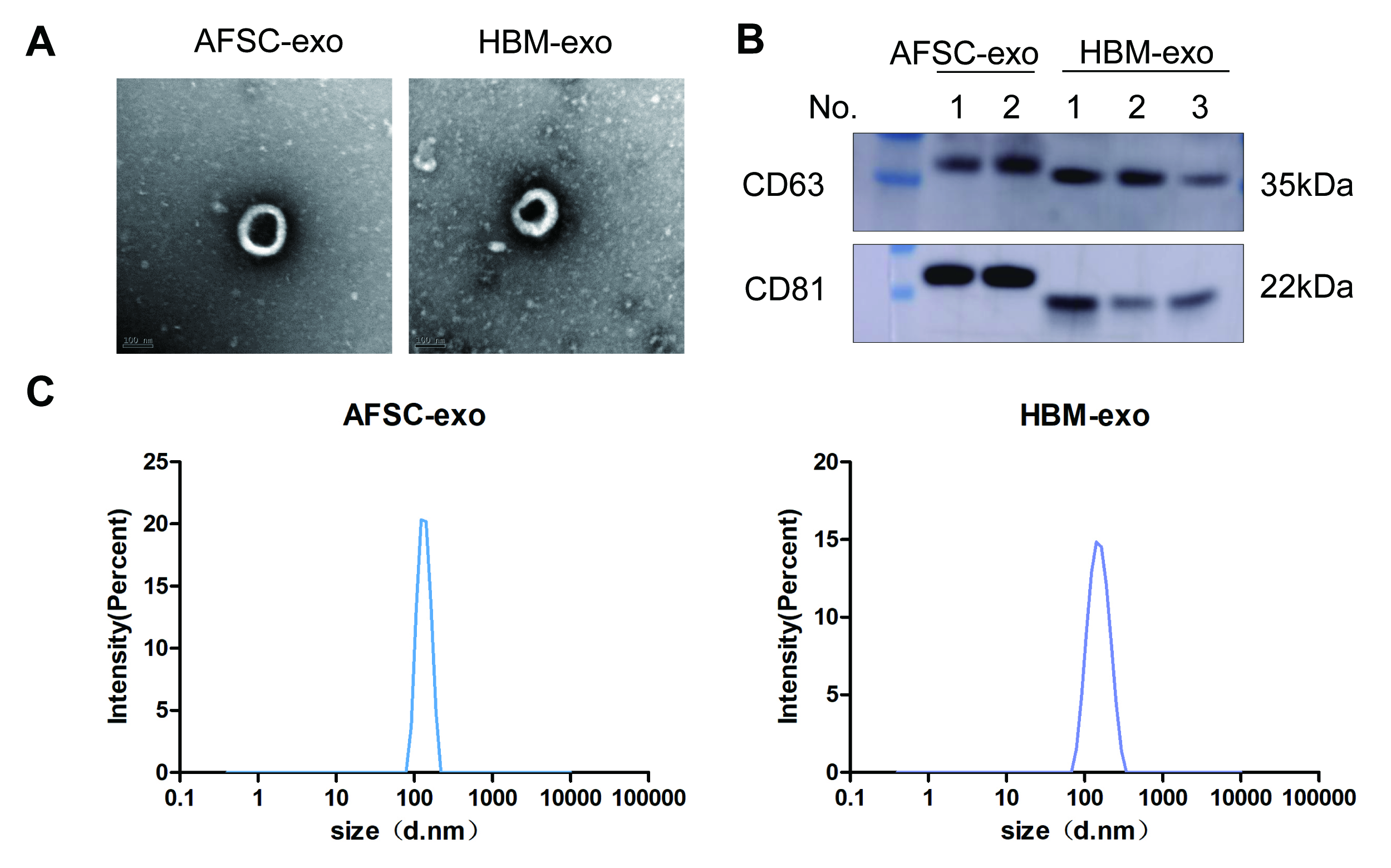

Supplement: Supplementary file 3 — High resolution image (TIF 18025 kb) [file 12015_2022_10470_MOESM2_ESM.tif]

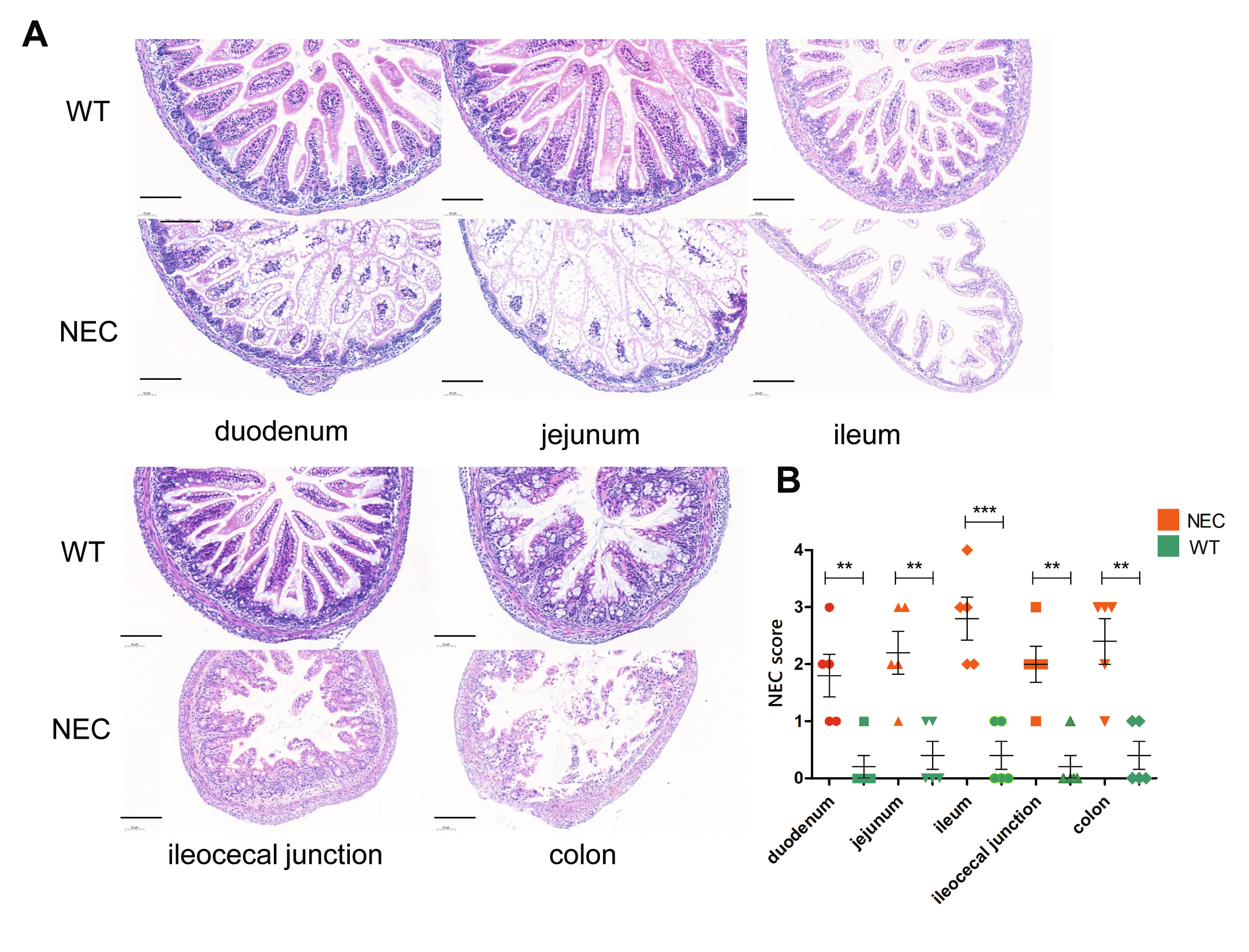

Supplement: Supplementary file 4 — (PNG 4417 kb) [file 12015_2022_10470_Fig7_ESM.png]

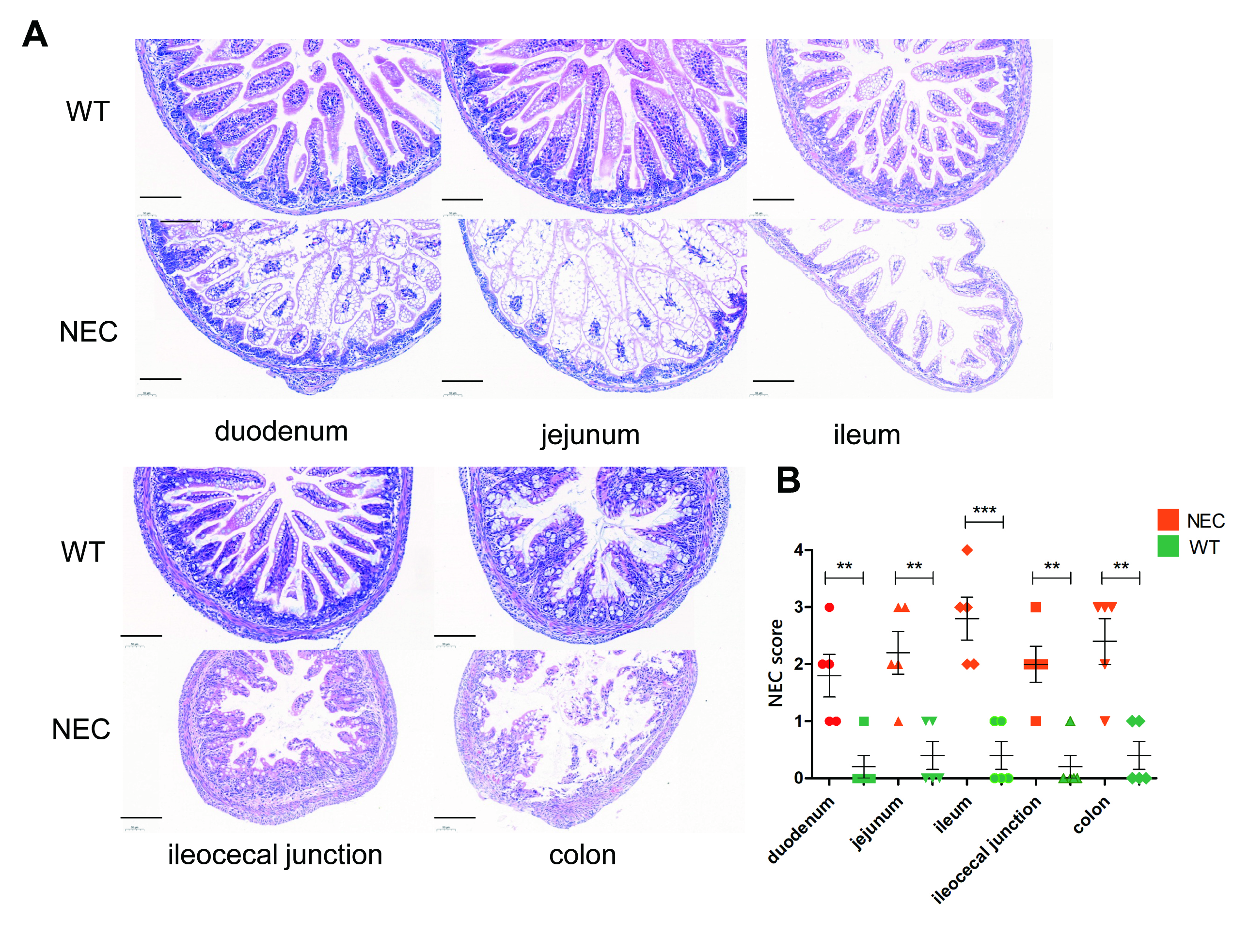

Supplement: Supplementary file 5 — High resolution image (TIF 26279 kb) [file 12015_2022_10470_MOESM3_ESM.tif]

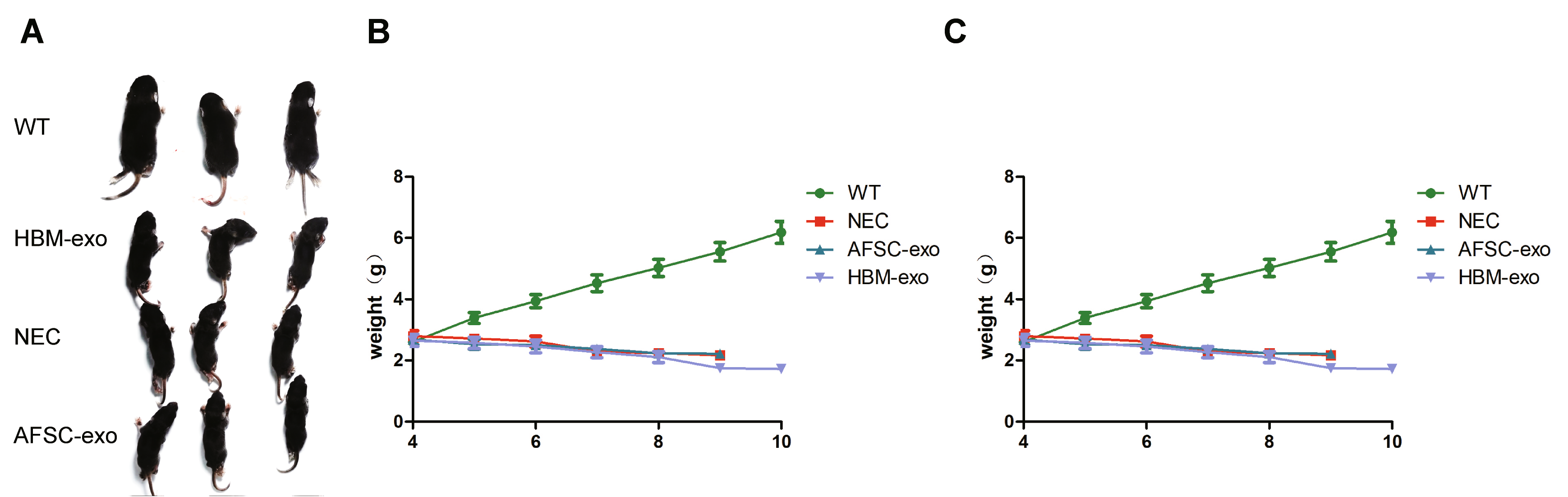

Supplement: Supplementary file 6 — (PNG 567 kb) [file 12015_2022_10470_Fig8_ESM.png]

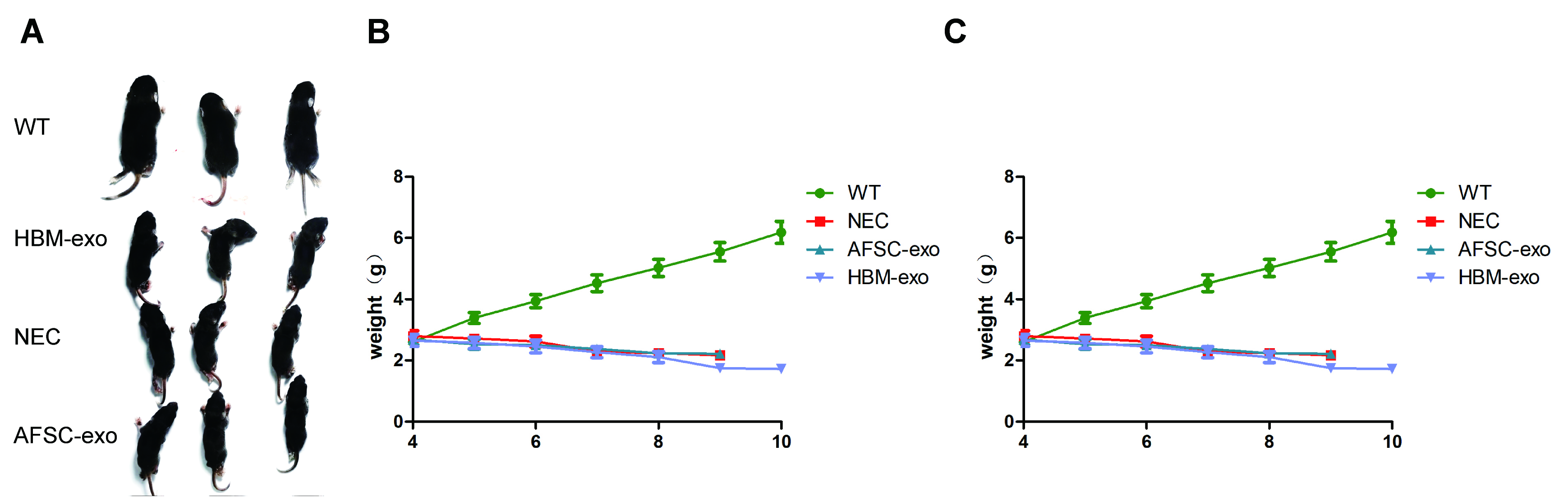

Supplement: Supplementary file 7 — High resolution image (TIF 16324 kb) [file 12015_2022_10470_MOESM4_ESM.tif]

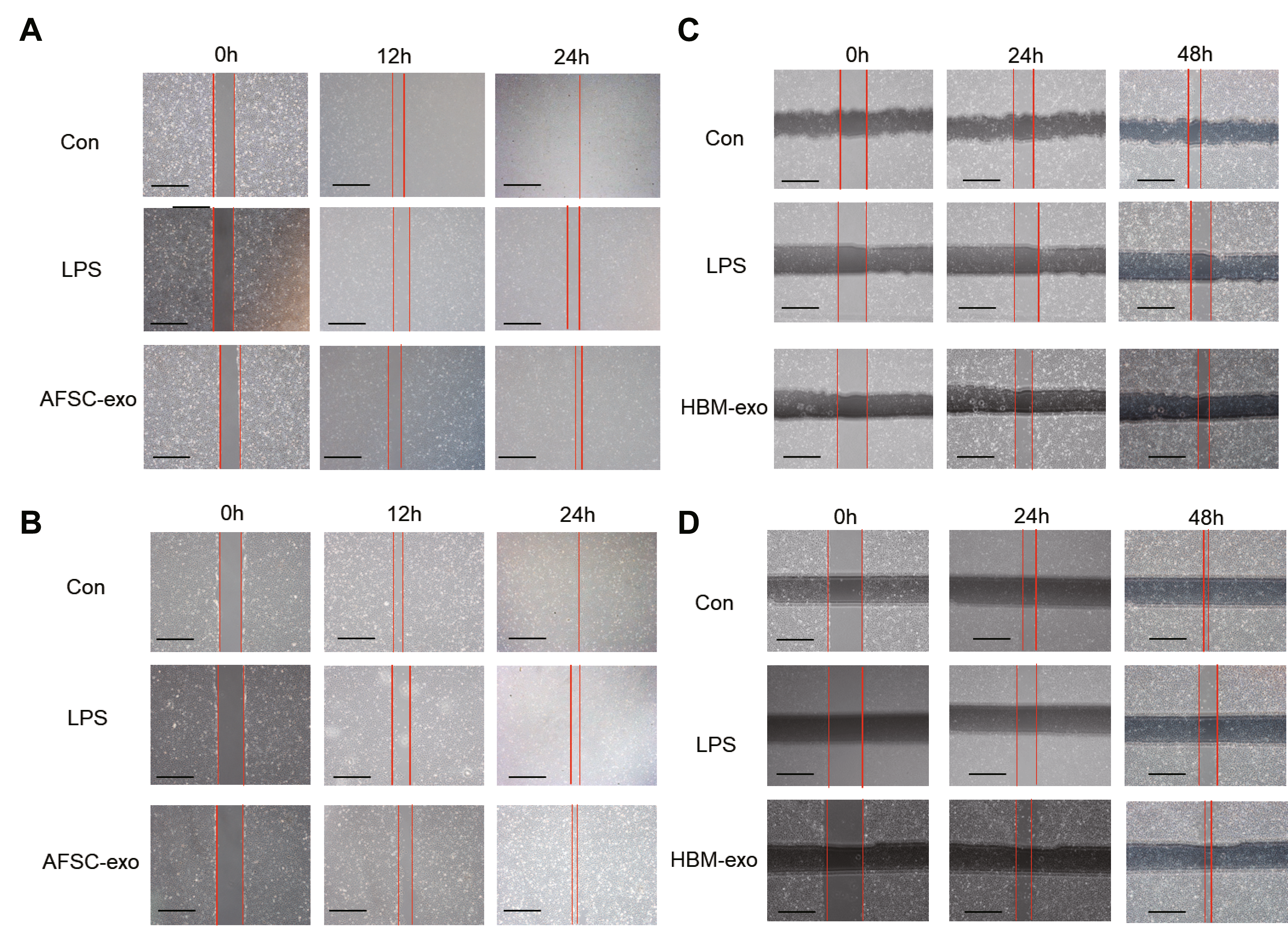

Supplement: Supplementary file 8 — (PNG 3607 kb) [file 12015_2022_10470_Fig9_ESM.png]

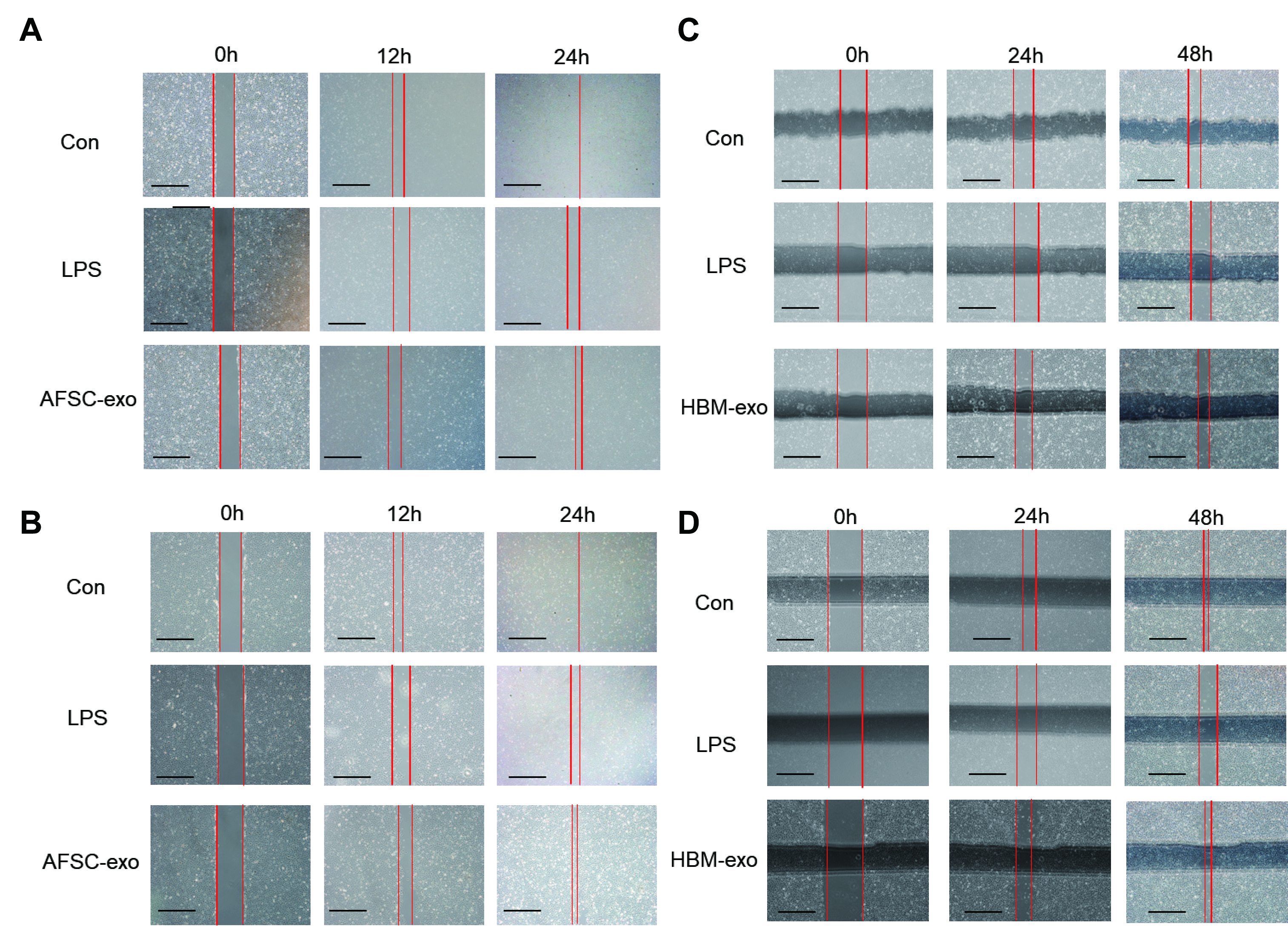

Supplement: Supplementary file 9 — High resolution image (TIF 38156 kb) [file 12015_2022_10470_MOESM5_ESM.tif]

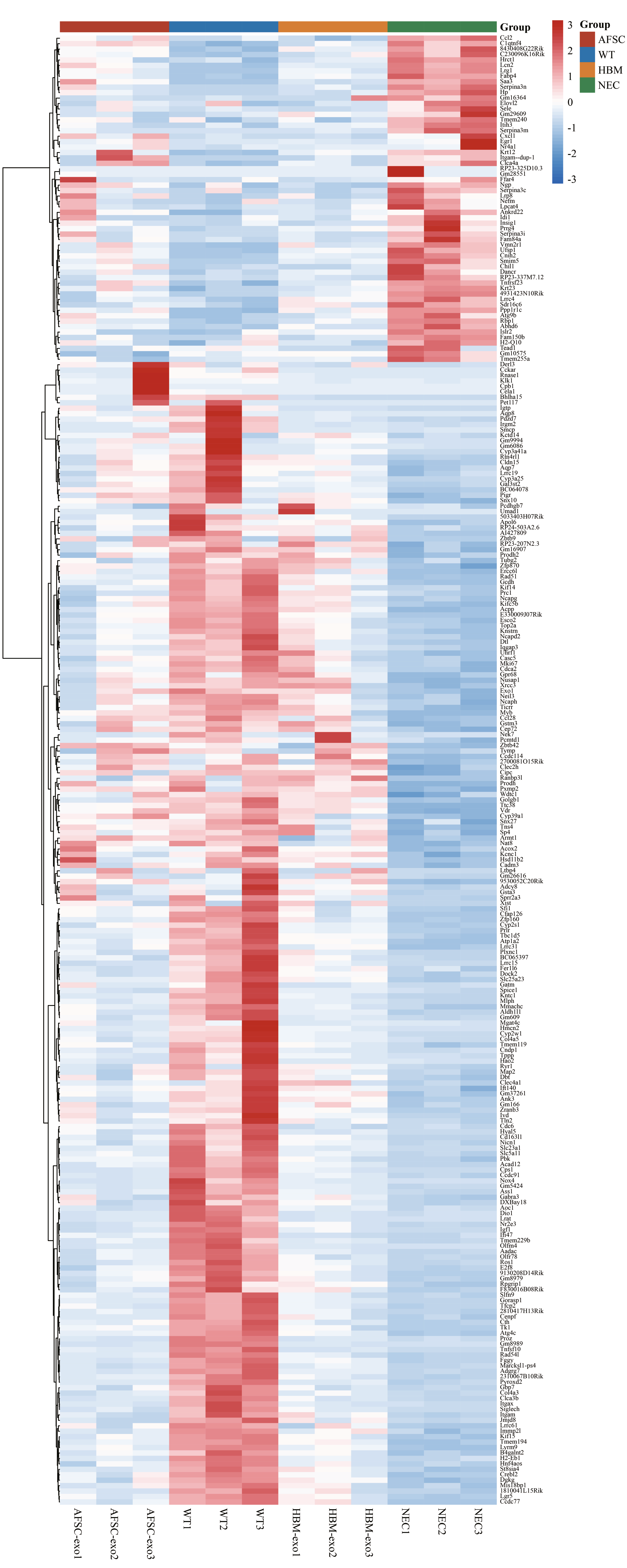

Supplement: Supplementary file 10 — (PNG 1326 kb) [file 12015_2022_10470_Fig10_ESM.png]

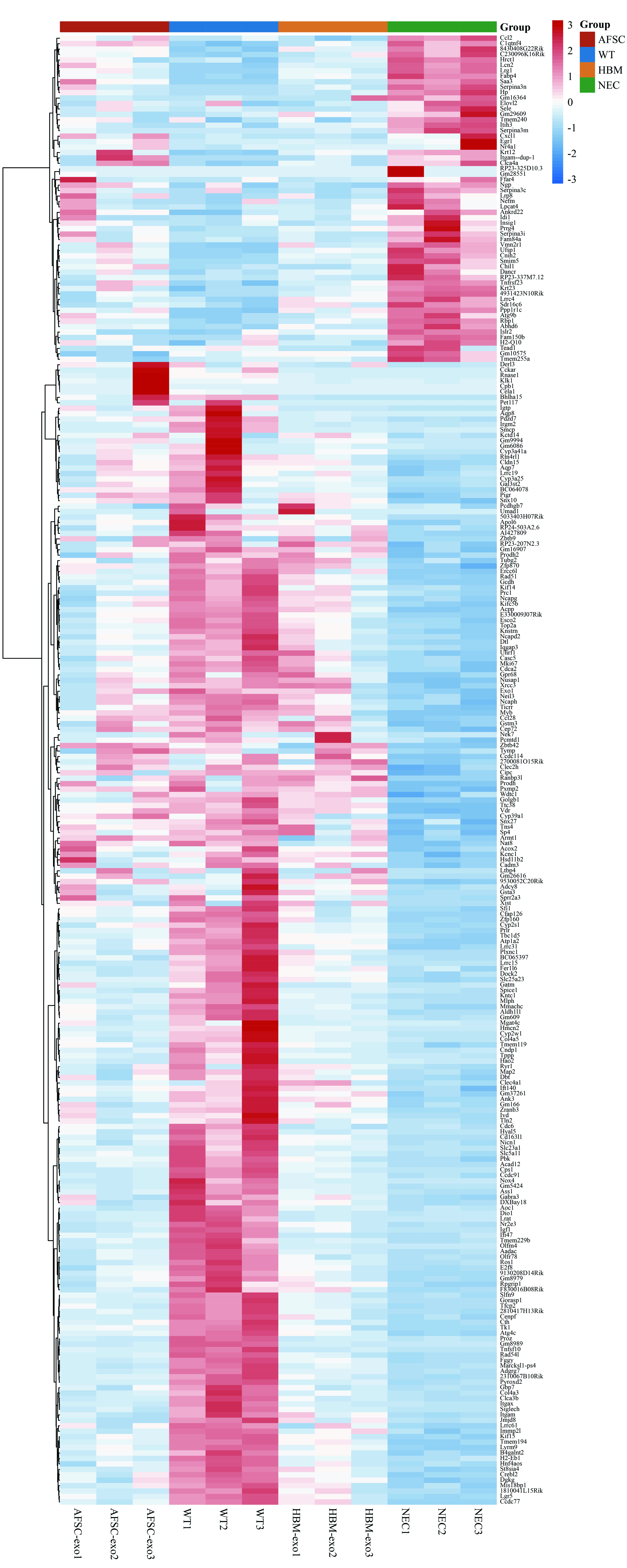

Supplement: Supplementary file 11 — High resolution image (TIF 60828 kb) [file 12015_2022_10470_MOESM6_ESM.tif]

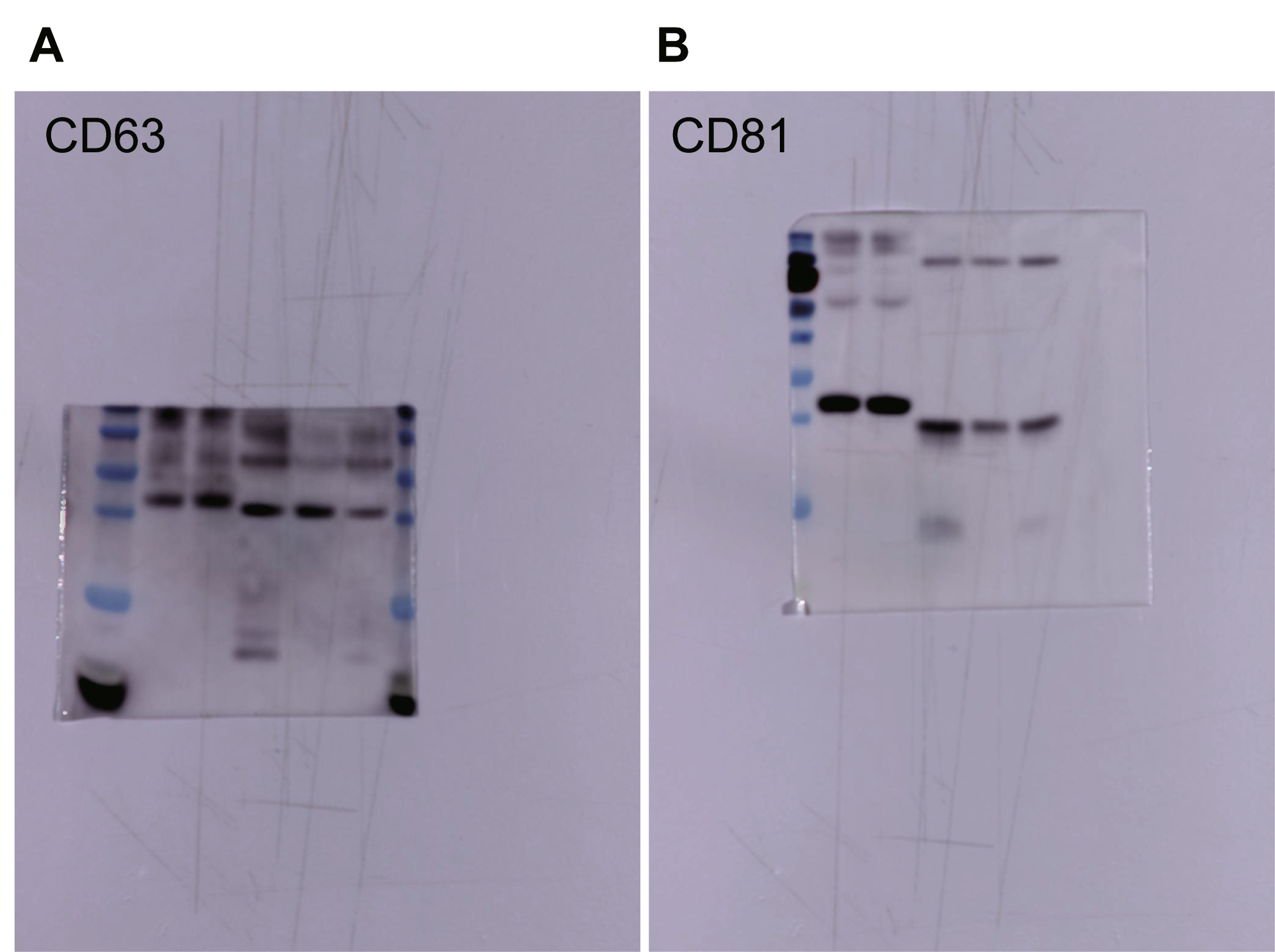

Supplement: Supplementary file 12 — (PNG 864 kb) [file 12015_2022_10470_Fig11_ESM.png]

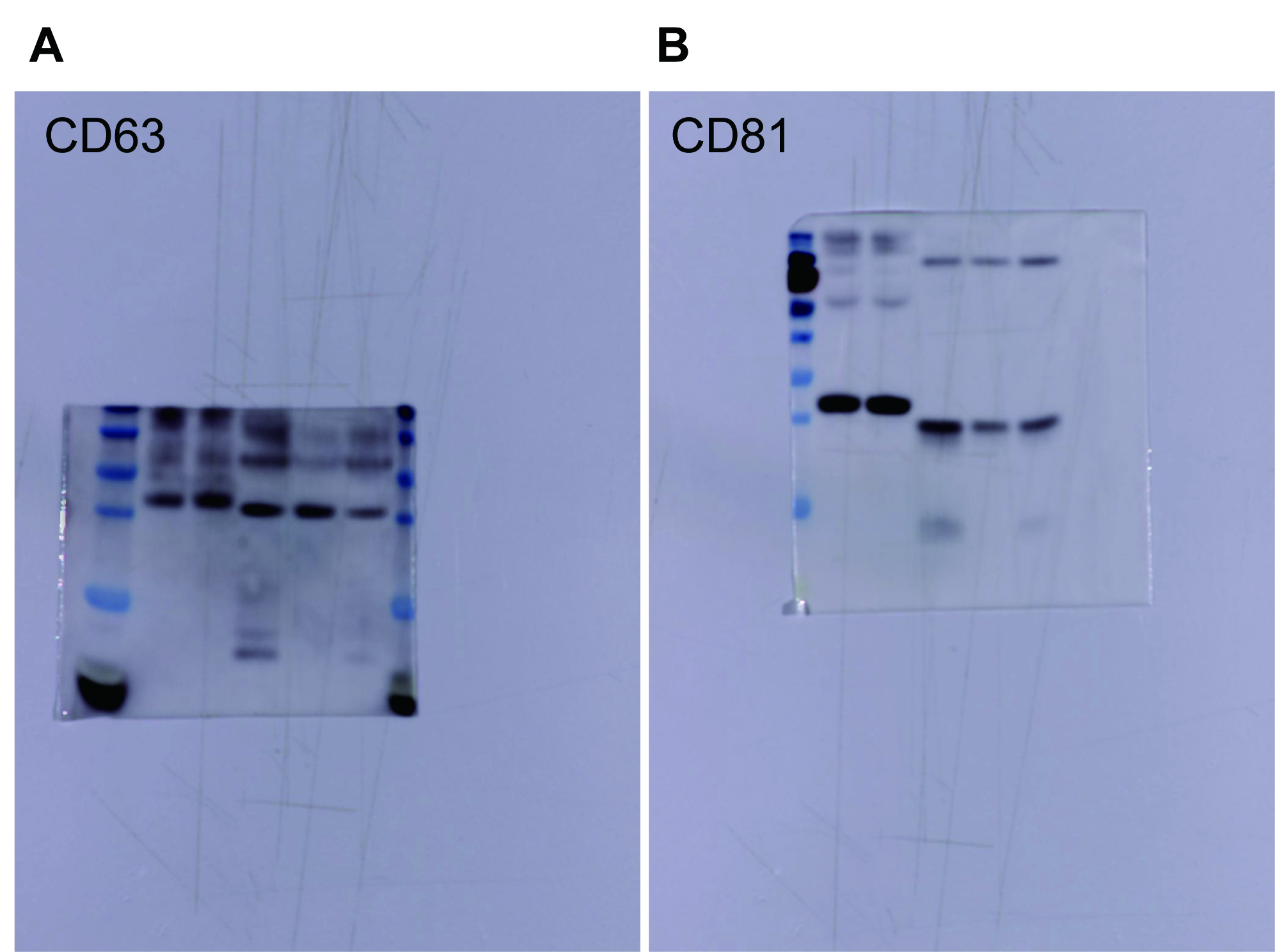

Supplement: Supplementary file 13 — High resolution image (TIF 15183 kb) [file 12015_2022_10470_MOESM7_ESM.tif]
